# Alisertib and Barasertib Induce Cell Cycle Arrest and Mitochondria-Related Cell Death in Multiple Myeloma with Enhanced Efficacy Through Sequential Combination with BH3-Mimetics and Panobinostat

**DOI:** 10.3390/cancers17142290

**Published:** 2025-07-09

**Authors:** Andrea Benedi, Manuel Beltrán-Visiedo, Nelia Jiménez-Alduán, Alfonso Serrano-Del Valle, Alberto Anel, Javier Naval, Isabel Marzo

**Affiliations:** Apoptosis, Immunity & Cancer Group, Instituto de Investigación Sanitaria-Aragón, University of Zaragoza, 50009 Zaragoza, Spain; a.benedi@unizar.es (A.B.); mbeltran@unizar.es (M.B.-V.); nj.alduan@gmail.com (N.J.-A.); 610927@unizar.es (A.S.-D.V.); anel@unizar.es (A.A.); jnaval@unizar.es (J.N.)

**Keywords:** Aurora kinase inhibitors, mitotic arrest, Bcl-2 proteins, cell death, multiple myeloma

## Abstract

Background: The treatment landscape for multiple myeloma (MM) has significantly evolved in recent decades with novel therapies like proteasome inhibitors, immunomodulatory drugs and monoclonal antibodies. However, MM remains incurable, necessitating new pharmacological strategies. Mitotic kinases, such as Aurora proteins, have emerged as potential targets. Selective inhibitors of Aurora A and B,- alisertib (MLN8237) and barasertib (AZD1152), respectively, have shown anti-myeloma activity in preclinical studies, with alisertib demonstrating modest efficacy in early clinical trials. Methods and Results: This study investigated the mechanisms of action of alisertib and barasertib and their combination with antitumor agents in a panel of five MM cells lines. Both drugs induced cell cycle arrest phase and abnormal nuclear morphologies. Alisertib caused prolonged mitotic arrest, whereas barasertib induced transient arrest, both resulting in the activation of mitotic catastrophe. These findings revealed three potential outcomes: cell death, senescence, or polyploidy. High mitochondrial reactive oxygen species (mROS) were identified as possible drivers of cell death. Caspase inhibition reduced caspase-3 activation but did not prevent cell death. Interestingly, alisertib at low doses remained toxic to Bax/Bak^DKO^ cells, although mitochondrial potential disruption and cytochrome c release were observed. Sequential combinations of high-dose Aurora kinase inhibitors with BH3-mimetics, and in specific cases with panobinostat, showed a synergistic effect. Conversely, the simultaneous combination of alisertib and barasertib showed mostly antagonistic effects. Conclusions: Alisertib and barasertib emerge as potential in vitro candidates against MM, although further studies are needed to validate their efficacy and to find the best combinations with other molecules.

## Simple Summary

Multiple myeloma (MM) remains a challenging disease despite significant therapeutic advances achieved with the introduction of novel therapies, prompting the search for new agents with unique and targeted mechanisms of action. Here, we investigated the selective Aurora kinase inhibitors alisertib (Aurora A) and barasertib (Aurora B) as potential anti-myeloma candidates. Our results demonstrate that both drugs exhibit anti-myeloma activity in vitro, and their sequential combination with BH3-mimetics and panobinostat could be beneficial. Therefore, these findings open the door to further explore selective inhibition of Aurora proteins as a targeted therapy in MM.

## 1. Introduction

Multiple myeloma (MM) is a complex and heterogeneous disease that represents around 10% of all hematological malignancies, being the second most frequent blood cancer in Western countries [1]. MM is characterized by the uncontrolled proliferation of malignant clonal plasma B cells chiefly localized in the bone marrow, being able to spread to other extramedullary areas through the bloodstream in the late stages of the disease [2]. These defective cells tend to secrete a monoclonal immunoglobulin (Ig) protein, known as M protein, which can be detected in both blood and urine and whose accumulation contributes to organ failure and the onset of early symptoms [3].

There have been many advances in relation to the treatment of MM in the last few decades. In fact, the recent Food and Drug Administration (FDA) approval of new, more selective agents such as proteasome inhibitors (bortezomib, ixazomib and carfilzomib), immunomodulatory drugs (IMiDs: thalidomide, pomalidomide and lenalidomide) and monoclonal antibodies (daratumumab, elotuzumab and isatuximab) as single agents or combined with traditional therapies based on alkylating agents, corticosteroids, anthracyclines and autologous stem cell transplantation (ASCT) has broadened the therapeutic arsenal to deal with the disease [1,4]. Unfortunately, even though novel agents have contributed to a significant increase in the overall survival of patients, MM is still an incurable malignancy. Furthermore, most patients eventually develop resistance (relapse/refractory MM) to the different lines of treatment, which is associated with poor prognosis [3,4]. All this reveals the urgent need to explore new specific strategies to fight MM.

Aurora proteins, a three-member family of serine/threonine kinases, play a pivotal role in the evolution of the cell cycle from the G2 phase to cytokinesis [5]. Mammalian cells express three highly conserved isoforms, Aurora A, B and C, which differ in subcellular locations and functions [5]. All of them are greatly expressed and activated during mitosis but they rapidly recover their basal levels at the end of this stage [6]. Aurora A is located in centrosomes and spindle microtubules and ensures suitable maturation and separation of centrosomes besides accurate mitotic spindle assembly and alignment of the metaphase chromosomes [6]. Aurora B binds to centromeres in the prometaphase and to the central zone of the mitotic spindle throughout anaphase and cytokinesis [7]. Moreover, it is the catalytic component of the chromosomal passenger complex (CPC) that regulates the spindle assembly checkpoint (SAC) and the correct microtubule/kinetochore association, chromosome segregation and cytokinesis [8]. Less well known is Aurora C, which is involved in germ cell meiosis and in preimplantation embryo development [9]. Therefore, dysregulation of these proteins results in centrosome amplification, aneuploidy, and genetic and chromosomal instability that can lead to a malignant transformation [10]. Interestingly, Aurora A and B have been found to be overexpressed in both solid tumors and hematologic malignancies [11,12,13,14,15], including MM, which correlates with drug resistance and unfavorable prognosis [16,17].

All this has triggered the development of Aurora protein inhibitors, some of them reaching clinical trials. Several of these compounds are directed against more than one Aurora family member, but specific inhibitors have also emerged, such as MK8745 [18] and alisertib (MLN8237) [19] or barasertib (AZD1152) [20] and BI 811283 [21], Aurora A or B inhibitors, respectively. Since one of the hallmarks of MM is genetic instability with numerous chromosomal abnormalities [22], these molecules may entail therapeutic benefit. Importantly, high levels of Aurora kinases have been correlated with poor prognosis in MM [17], and genetic inhibition of these proteins has been shown to reduce proliferation and resistance to anti-myeloma drugs [23,24,25].

Alisertib is a small second-generation orally bioavailable molecule much more selective for Aurora A (IC_50_ = 1.2 nM) than Aurora B (IC_50_ = 396.5 nM) [19]. Alisertib assembly to the ATP-binding site prevents autophosphorylation at Thr288 and thus Aurora A activation [26]. This inhibition delays entry into mitosis and induces mitotic spindle and chromosome alignment blunders, provoking cell cycle arrest at the G2/M phase, aneuploidy, polyploidy, mitotic catastrophe, and finally senescence, re-entry to the cell cycle or cell death [26,27,28]. Recently, alisertib has been reported to up-regulate p53 the pathway, which undergoes mitotic arrest, and activate the mitochondrial apoptotic pathway to prompt apoptosis [27,29,30].

Barasertib is a much more potent and selective ATP-competitive inhibitor of Aurora B (Ki = 1 nM) compared to Aurora A (Ki = 1.4 µM), although it also inhibits 50 other kinases [20,31]. Conceived as a pro-drug unable to cross cell membranes, it is rapidly converted into its more active moiety (barasertib-hQPA) after in vivo administration [32]. Aurora B contains the Glu177 residue, which binds to barasertib by hydrogen bonding, while in Aurora A this residue is replaced by a threonine (Thr217), which accounts for its selectivity [20]. More recently, the Arg159 residue of Aurora B and binding interactions in the rear hydrophobic pocket have been proposed to be crucial for the selectivity of barasertib [33]. At the cellular level, it induces chromosome misalignment and blocks cytokinesis, but allows for endoreduplication, which prompts the generation of multinucleated giant cells with a DNA content equal to or greater than 4n (polyploidy) that can perish or enter into a senescent state [34]. Nevertheless, our knowledge of the molecular pathways involved in cell changes induced by Aurora kinase inhibitors is limited.

A large number of in vitro and in vivo studies have highlighted alisertib activity as a single agent against a wide range of tumors, including MM [30,35,36,37,38]. Likewise, barasertib has shown antitumor potential in AML (acute myeloid leukemia) cell lines and several murine xenograft studies [39]. Additionally, combination with other therapies enhances their antitumoral effects in different preclinical models [40,41,42,43,44]. These results have supported their inclusion in multiple clinical trials in solid and blood cancers (MM inclusive), but the data reveal rather modest efficacy [32,36,45]. Although this has not yet led to their clinical approval, both drugs continue to be tested in solid and hematological cancers, either as monotherapies or in combination with other treatments. In fact, potentially synergistic combinations with other agents may help enhance their efficacy.

Dysregulation of apoptosis, caused by an imbalance in interactions between the Bcl-2 family proteins, is a typical attribute of cancer cells [46]. In these cells, anti-apoptotic members (Bcl-2, Bcl-X_L_ and Mcl-1) sequester “BH3-only” proteins (e.g., Bim, PUMA, Noxa, Bid) by binding to their BH3 domain, thus avoiding Bax and Bak oligomerization and subsequent mitochondrial outer membrane permeabilization (MOMP) and caspase activation [47,48]. Due to the relevance of these interactions, many efforts have lately been devoted to create the so-called “BH3-mimetics”, small organic molecules that mimic the pro-apoptotic activity of the “BH3-only” proteins via interaction with the hydrophobic groove of anti-apoptotic proteins, managing to displace “BH3-only” members and reactivate the mitochondrial apoptotic pathway [47,48]. ABT-199 (venetoclax), a specific Bcl-2 inhibitor, has been shown to be effective as a single agent and combined with other therapies in different cancer types, including hematologic neoplasms such as leukemias and lymphomas [49,50,51]. Therefore, the FDA and the European Medicines Agency (EMA) rapidly approved its use for the treatment of chronic lymphocytic leukemias (CLLs), small lymphocytic lymphoma (SLL) and AML as monotherapy or in combination with other treatments [52]. A-1155463 is a highly potent and selective inhibitor of Bcl-X_L_ that displays cytotoxic activity against Bcl-X_L_-dependent cancer cell lines [46,53]. Moreover, it synergizes with venetoclax and docetaxel in solid tumor cell lines [54]. S63845 is an extremely potent and selective Mcl-1 inhibitor against tumors dependent on this protein in vitro and in vivo, including leukemias and MM [55]. In addition, its combination with bortezomib in MM cells produces a slight synergistic effect, while combination with kinase inhibitors is remarkably toxic in breast cancer, non-small-cell lung cancer (NSCLC) and melanoma cell lines [55,56]. The triple combination of venetoclax, S63845 and dexamethasone is strongly synergistic in vitro and excellently tolerated in an aggressive disseminated model of MM likewise [57].

Taking all this into account, the present study intends to provide insight into the mechanism of action of the Aurora A and B kinase inhibitors alisertib and barasertib and to evaluate their therapeutic potential alone and in combination with novel agents in MM cell lines, including the selective BH3-mimetics ABT-199, A-1155463 and S63845. We found that alisertib and barasertib induce mitotic arrest followed by cell death, entry into a senescent state or the induction of polyploidy through different mechanisms. Moreover, their sequential combination with BH3-mimetics and the histone deacetylase inhibitor panobinostat often results in synergism, paving the way for their potential use in certain situations, although further studies are needed.

## 2. Materials and Methods

### 2.1. Drugs and Reagents

Antimitotic drugs (alisertib and barasertib), BH3-mimetics (ABT-199, A-1155463, S63845 and ABT-737), the histone deacetylase inhibitor panobinostat, the RIPK1 inhibitor necrostatin-1 (Nec-1), and caspase inhibitors Z-VAD(OMe)-FMK, Z-IETD-FMK and Z-DEVD-FMK were purchased from MedChemExpress (Monmouth Junction, NJ, USA). The antioxidant glutathione (GSH) and poly-L-Lysine were purchased from Sigma-Aldrich-Merck (Darmstadt, Germany). Mitochondrial probes DiOC_6_(3) and TMRE were obtained from Sigma-Aldrich-Merck, while MitoSOX^TM^ and MitoTracker^TM^ Green FM were obtained from Thermo Scientific (Waltham, MA, USA). GSH stock solution and poly-L-Lysine solution were prepared in Milli-Q water at 100 mM and a 0.1 mg/mL concentration, respectively. The remaining stock solutions were made in DMSO (Sigma-Aldrich-Merck). The final DMSO concentration in all experiments was lower than 0.5%.

### 2.2. Cell Lines and Culture

The human MM cell lines NCI-H929 and U266 were kindly provided by Dr. Antonio Campos Caro (Hospital Puerta del Mar, Cádiz, Spain), RPMI 8226 was provided by Dr. Martine Amiot (CRCINA, Nantes, France), and OPM-2 and MM.1S were obtained from the DSMZ (Braunschweig, Germany) and the ATCC (Manassas, VA, USA), respectively. These cell lines have been previously reported to express both Aurora A and Aurora B [17,23,24,58,59]. MM.1S Ø (transfected with empty vector), MM.1S Bax^KO^, MM.1S Bak^KO^ and MM.1S Bax^/^Bak^DKO^ [60] were generated in our laboratory. The human pro-monocytic leukemia cell line U937 was kindly provided by Dr. Jeremy Brock (University of Glasgow, Glasgow, UK), and the U937 ρ^0^ cell subline (lacking mtDNA) was obtained in our laboratory as described by Gamen et al. [61]. All cell lines were verified by STR analysis, and the absence of mycoplasma contamination was periodically checked using the commercial kits Mycoplasma Gel Detection Kit (Biotools, Singapore) or MycoStrip^TM^ (InvivoGen, San Diego, CA, USA) following the manufacturers’ instructions. MM and U937 cells were routinely cultured in RPMI 1640 (1×) + GlutaMAX^TM^ medium (Gibco; Waltham, MA, USA) supplemented with 10–15% fetal bovine serum (hereafter, FBS, Sigma-Aldrich-Merck; MO, USA) and 1% of the antibiotics penicillin (100 U/mL) and streptomycin (100 µg/mL) (PAN-Biotech; Bayern, Germany) (henceforth, complete medium). U937 ρ^0^ cells were cultured in RPMI 1640 (1x) + GlutaMAX^TM^ medium supplemented with 10% FBS, glucose (PanReac; Barcelona, Spain, 2.5 mg/mL), pyruvate (PanReac, 100 µg/mL), uridine (Sigma-Aldrich-Merck, 50 µg/mL) and antibiotics. All cell lines were grown at 37 °C with 5% CO_2_ and humidified air. Cell viability was evaluated by using the Trypan-blue exclusion test to start the experiments.

### 2.3. Proliferation and Cytotoxicity Assays

The MTT [3-(4,5-dimethylthiazol-2-yl)-2,5-diphenyltetrazolium bromide] assay was performed as previously described [62]. For cytotoxicity assays, cells were seeded in complete medium in flat-bottom, 24- or 48-well plates and treated with antimitotic drugs, BH3-mimetics or multiple combinations during the indicated times for each condition. When indicated, cells were preincubated for at least 1 h with the caspase inhibitors Z-VAD, Z-IETD, Z-DEVD and/or Nec-1 (30 µM) prior to the addition of the drugs, and the inhibitors were refreshed every 24 h. Apoptosis and necrosis were determined by flow cytometry, by measuring phosphatidylserine (PS) exposure on the outer leaflet of the plasma membrane, and cell membrane permeabilization via staining with 50 µL of a mixture of 1× Annexin V Binding Buffer (1× ABB; 140 mM NaCl, 2.5 mM CaCl_2_, 10 mM HEPES/NaOH pH 7.4 in sterile Milli-Q water), Annexin V conjugated with FITC or DY634 and, when indicated, 7-amino-actinomycin D (7-AAD) and 10 min of incubation in the dark at room temperature (RT). Recombinant Annexin V was obtained and conjugated with FITC or DY634 in our laboratory as described by Logue et al. [63].

### 2.4. Cell Cycle Analysis and BrdU Assays

Cells were washed twice with PBS after treatments, fixed with cold 70% (*v*/*v*) ethanol by vortexing and stored at −20 °C for at least 24 h. Cells were then washed with cold PBS and stained with 500 µL of a propidium iodide (PI) and RNase (Immunostep; Salamanca, Spain) solution for 20 min at RT in darkness for analysis by flow cytometry. DNA synthesis was analyzed by flow cytometry using the BrdU (5-bromo-2-deoxyuridine) Flow Kits (BD Pharmingen; San Diego, CA, USA) following the manufacturer’s instructions.

### 2.5. Cytochrome c Release

After treatments, cells were washed with PBS and resuspended in 200 µL of permeabilization buffer (50 µg/mL digitonin and 10 mM KCl in PBS pH 7.4). In the case of positive controls, they were resuspended in cold PBS. All samples were incubated for 5 min on ice, diluted in PBS and fixed with a 4% paraformaldehyde (PFA) solution for 15 min at RT. Next, cells were rinsed and incubated in 100 µL of a blocking solution (0.05% (*w/v*) saponin and 3% (*w*/*v*) BSA in PBS pH 7.4) for 1 h at RT. Subsequently, samples were incubated with a mouse anti-cytochrome c antibody (clone 6H2.B4, #556432, BD Pharmingen) or a mouse anti-IgGκ antibody for isotype control (clone 107.3, #554721, BD Biosciences; San Jose, CA, USA) overnight at 4 °C in agitation. Then, samples were diluted in PBS, washed, resuspended in 50 µL of blocking buffer containing an Alexa 488-conjugated mouse anti-IgG (H+L) antibody (#A11029, Thermo Scientific) diluted 1:1000 and were incubated for 30 min at RT in the dark. Finally, cells were resuspended in 300 µL of PBS for analysis by flow cytometry.

### 2.6. Caspase-3 Activation

Cells (10^6^ per point) were rinsed with PBS and fixed with 4% PFA for 15 min. Next, they were washed again, resuspended in permeabilization buffer (0.1% (*w*/*v*) saponin in PBS pH 7.4) and incubated for 20 min at RT. Subsequently, cells were rinsed again and incubated with an FITC-conjugated rabbit anti-active caspase-3 antibody (#559341, BD Pharmingen) diluted 1:20 in PBS supplemented with 5% FBS for 30 min at RT in darkness. Finally, cells were washed once again before flow cytometry analysis.

### 2.7. Analysis of Mitochondrial Transmembrane Potential, Mitochondrial Mass Assessment and Mitochondrial ROS Generation

Mitochondrial transmembrane potential was evaluated by flow cytometry with the mitochondrial probes DiOC_6_(3) or TMRE at a final concentration of 10 nM or 60 nM, respectively, for 30 min at 37 °C in darkness. In some experiments, mitochondrial probes were used simultaneously with Annexin V-DY634. In this case, cells were first stained with DiOC_6_(3) or TMRE in 1× ABB for 30 min at 37 °C and subsequently with DY634-conjugated Annexin V for 10 min at RT in the dark. Stained cells were analyzed by flow cytometry.

For mitochondrial mass and mitochondrial ROS, cells were resuspended in 100 µL of complete medium containing 200 nM MitoTracker^TM^ Green FM or 5 µM MitoSOX, respectively, and incubated for 30 min at 37 °C in the dark before flow cytometry analysis.

### 2.8. Flow Cytometry

A total of 10,000 cells were acquired on a FACSCalibur^TM^ flow cytometer (BD Biosciences), and data were independently collected with CellQuest Pro Version 6.0 (BD Biosciences) and processed with FlowJo 10.8.1 (Becton Dickinson* (BD)*; Franklin Lakes, NJ, USA) and GraphPad Prism Version 10.5.0; (San Diego, CA, USA) software.

### 2.9. Nuclei Staining

Cells were treated in flat-bottom, 24- or 48-well plates (500 µL/well) with 100 nM alisertib and 5 µM barasertib for 24–72 h. After treatments, cell nuclei were stained with Hoechst 333242 (2 µg/mL) (Molecular Probes; Eugene, OR, USA) for 15 min in the dark at RT, and then cells were washed with PBS and transferred to sterile flat-bottom, 24-well plates containing poly-L-lysine-coated coverslips. Plates were centrifugated at 2850× *g* for 3 min and cells were fixed with 4% PFA for 15 min at RT. Then, cells were washed and coverslips were recovered, which were then mounted onto a drop of Fluoromount-G (SouthernBiotech; Birmingham, AL, USA) on slides, and the preparations were sealed and stored at 4 °C in darkness. Nuclear morphology was visualized in a fluorescence microscope (Eclipse 50i, Nikon; Tokyo, Japan) coupled to digital photography equipment (DXM1200F, Nikon), and images were taken with the Nikon ACT-1 version 2.62 software (Nikon).

### 2.10. Immunofluorescence

MM.1S wild-type and Bax^/^Bak^DKO^ cells (10^5^/mL) were seeded in flat-bottom, 24-well plates (1 mL/well) containing poly-L-lysine-pretreated coverslips and incubated with 100 nM alisertib for 48 h. After treatments, nuclei were stained with a Hoechst 33342 probe (2 µg/mL) for 15 min in the dark at RT, and then plates were centrifuged at 2850× *g* for 3 min. Cells were then fixed with 2% PFA in RPMI 1640 complete medium at 37 °C for 15 min and washed with PBS. Afterwards, cells were incubated with a mixture of 0.1% saponin in PBS, goat serum (1:20) and anti-cytochrome c antibody (1:200) in a wet chamber for 1 h at RT. Cells were then washed three times with 0.1% saponin in PBS and incubated with a mixture of 0.1% saponin in PBS, goat serum (1:20) and anti-mouse Alexa Fluor 488 antibody (Invitrogen A-11029, 1:1000) for 1 h at RT. After this, cells were washed three times with 0.1% saponin in PBS, PBS and distilled water, in this order. Finally, coverslips were recovered, mounted in a drop of Fluoromount-G on slides, and the samples were sealed and stored at 4 °C in the dark. Preparations were observed under a confocal fluorescence microscope (Zeiss LM 880, Zeiss; Oberkochen, Germany), and images were taken with the Zen Black (Zeiss) software, which were analyzed and processed with Zen Blue 2.3 SP1 FP3 (Zeiss) and ImageJ2 version 2.14.0 (National Institutes of Health; Bethesda, MD, USA).

### 2.11. Senescence-Associated β-Galactosidase (SA β-gal) Activity

After treatments, cells were resuspended in fresh medium and kept in culture for 5 days, then transferred to 24-well plates containing sterile coverslips pretreated with poly-L-lysine. Cells were washed with PBS and fixed with a solution of 2% (*v*/*v*) PFA and 0.2% (*v*/*v*) glutaraldehyde in PBS for 10 min at 4 °C. Subsequently, they were rinsed and incubated overnight at 37 °C with the SA β-gal staining solution (5 mM K_3_[Fe(CN)_6_], 5 mM K_4_[Fe(CN)_6_]·3H_2_O and 2 mM MgCl_2_ in PBS pH 6.0) and X-Gal substrate (1 mg/mL) in the absence of gas exchange. Finally, cells were observed under an inverted microscope (Eclipse TE300, Nikon) 24 h after staining and images were collected with the aforementioned digital photography equipment and software.

### 2.12. Immunoblotting

Total protein cell lysates were prepared by resuspending 3 × 10^6^ cells in 60 µL of lysis buffer (50 mM Tris-HCl pH 7.6 buffer containing 1% (*v*/*v*) Triton X-100, 10% (*v*/*v*) glycerol, 150 mM NaCl, 1 mM EDTA, 1 mM Na_3_VO_4_, 10 mM Na_4_PO_7_, 10 µg/mL leupeptin, 10 mM NaF, 1 mM phenylmethylsulfonyl fluoride [PMSF] and a cOmplete^TM^ protease inhibitor cocktail tablet [11697498001, Roche; Basel, Switzerland]) and incubating them on ice for at least 30 min. Solubilized proteins were resolved by SDS-10/15% PAGE and transferred to nitrocellulose membranes. The latter were blocked with 5% skimmed milk powder dissolved in buffer B (0.1% (*w*/*v*) Tween-20 and (1 g/L) thimerosal dissolved in PBS pH 7.4) for a minimum of 30 min and incubated with the suitable primary antibodies diluted in antibody solution buffer (5% (*w*/*v*) BSA and 0.05% (*w*/*v*) NaN_3_ dissolved in buffer B) for two hours at RT or overnight at 4 °C. The following specific antibodies against human proteins were used were: mouse anti-BURB1 (612503, BD Biosciences), mouse anti-cyclin B1 (sc-245, Santa Cruz Biotechnology; St, Dallas, TX, USA) and mouse anti-MAD2 (610678, BD Biosciences). After incubation, membranes were washed with buffer B and incubated for 60–90 min at RT with a goat anti-mouse IgG-peroxidase secondary antibody (A9169/A9044) (Sigma-Aldrich-Merck) diluted in buffer B with 2.5% milk . Membranes were washed again and incubated with the peroxidase substrate [Pierce^TM^ ECL Western Blotting substrate kit (Thermo Fisher Scientific)]. Bands were detected by using Amersham^TM^ Imager 600 equipment (*G*E Healthcare; Chicago, IL, USA). β-actin was used as a protein loading control (mouse anti-β-actin, A-1978, Sigma-Aldrich-Merck) for band quantification with ImageJ software.

### 2.13. Statistical Methods

All statistical analyses were performed by using GraphPad Prism 8.0.1 software. For quantitative variables, the results are shown as the mean ± standard deviation (SD) and the confidence interval (* *p* < 0.05, ** *p* < 0.01, *** *p* < 0.001, **** *p* < 0.0001). As indicated in each case, data were analyzed using the following statistical tests: two-tailed unpaired *t*-test for the analysis of two variables of independent samples; two-tailed paired *t*-test for the analysis of two variables in matching or paired samples; one-way ANOVA or two-way ANOVA with Dunnett’s post-test to compare two or three variables, respectively, between two or more independent groups with respect to a control group; one-way ANOVA with Sidak’s post-test to compare two variables between pre-selected independent groups; two-way ANOVA with Sidak’s post-test to compare three variables between two independent groups; two-way ANOVA with Tukey’s post-test to compare three variables between three or more independent groups. The Bliss independent model as described in [64] was used to identify potential synergistic combinations in MM cell lines.

## 3. Results

### 3.1. Aurora Kinase Inhibitors Display Antitumor Potential in MM Cell Lines

**Figure 1:**
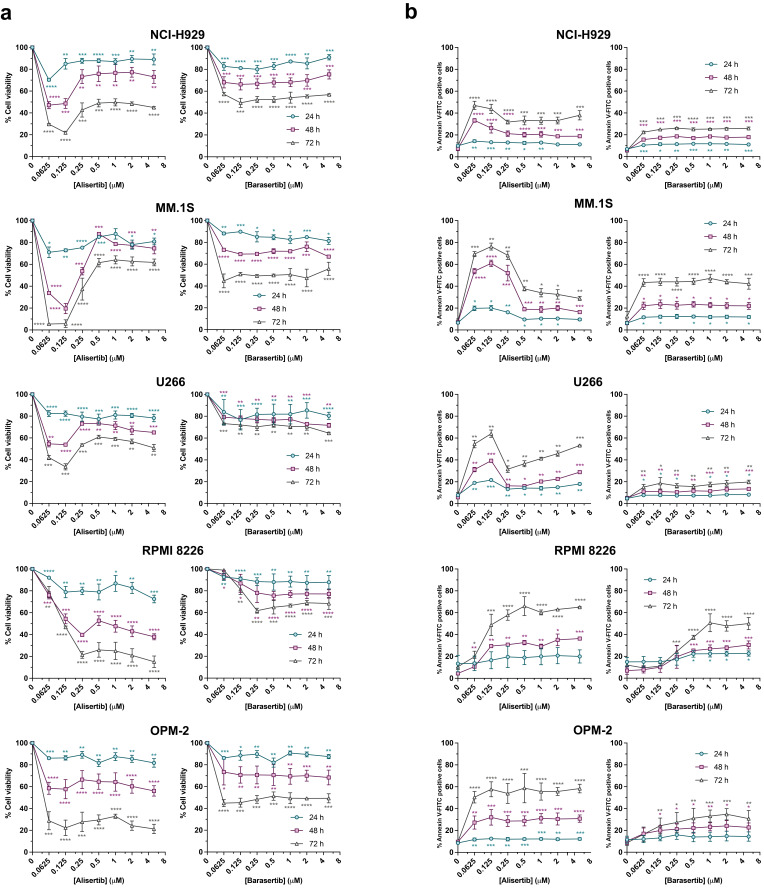
Analysis of the growth inhibitory (**a**) and cell-inducing potential (**b**) of alisertib and barasertib in MM cells. A panel of five MM cell lines were treated with increasing concentrations of alisertib and barasertib (0–5 µM) for 24–72 h. (**a**) Cells were seeded at 1.5–3 × 10^5^ cells/mL in 96-well plates with increased concentrations of alisertib (n = 2–5) and barasertib (n = 2–6). At 24, 48 or 72 h, an MTT assay was performed. (**b**) Cells were seeded in 24- or 48-well plates at 1.5–3 × 10^5^ cells/mL with increasing concentrations of alisertib or barasertib. Apoptosis was determined at 24, 48 and 72 h by measuring PS exposure through the binding of Annexin V-FITC and flow cytometry (n = 2–5, alisertib; n =2–8, barasertib). The global mean and SD are shown. Two-tailed unpaired t-test statistical analysis was performed, comparing each concentration to controls for each time. * *p* < 0.05; ** *p* < 0.01; *** *p* < 0.001; **** *p* < 0.0001.

First, the antiproliferative activity of alisertib and barasertib was evaluated on a panel of human MM cell lines. Both antimitotics inhibited cell growth in a dose- and time-dependent way in all cell lines (Figure 1a). Interestingly, when cells were incubated with alisertib, two distinct response profiles were observed. U266, NCI-H929 and, in particular, MM.1S cells were more sensitive to low doses than to high doses of the inhibitor, with a maximum inhibitory effect in the range 62.5–125 nM in all three cell lines. In contrast, the proliferative capacity of RPMI 8226 and OPM-2 cells remarkably decreased from 48 h of incubation at concentrations of 250 nM (RPMI 8226) or 62.5 nM (OPM-2).

The toxicity of both inhibitors was also time-dependent, as Annexin V binding analysis revealed (Figure 1b). Coincidentally, the pattern observed in cell proliferation assays correlates with the apoptotic levels induced by alisertib at 48 and 72 h, reaching a maximum at concentrations of 62.5–125 nM in NCI-H929, MM.1S and U266 cells, but decreasing at higher concentrations. However, the mortality of U266 cells increased slowly and progressively from doses of 250 nM, in particular at 72 h. On the other hand, a ‘plateau effect’ was detected in RPMI 8226 and OPM-2 cells after 48 and 72 h of treatment, reaching around 60% cell death at 62.5 nM in OPM-2 cells and 250 nM in RPMI 8226 cells. The effect of barasertib on cell growth and survival was quite reduced even at 72 h in NCI-H929, U266 and OPM-2 cells, while MM.1S and RPMI 8226 cells were more sensitive to prolonged treatments, with the highest apoptotic rates in both cell lines being equivalent (40–50%).

### 3.2. Aurora Kinase Inhibitors Induce Mitotic Arrest and Senescence in MM Cell Lines

**Figure 2:**
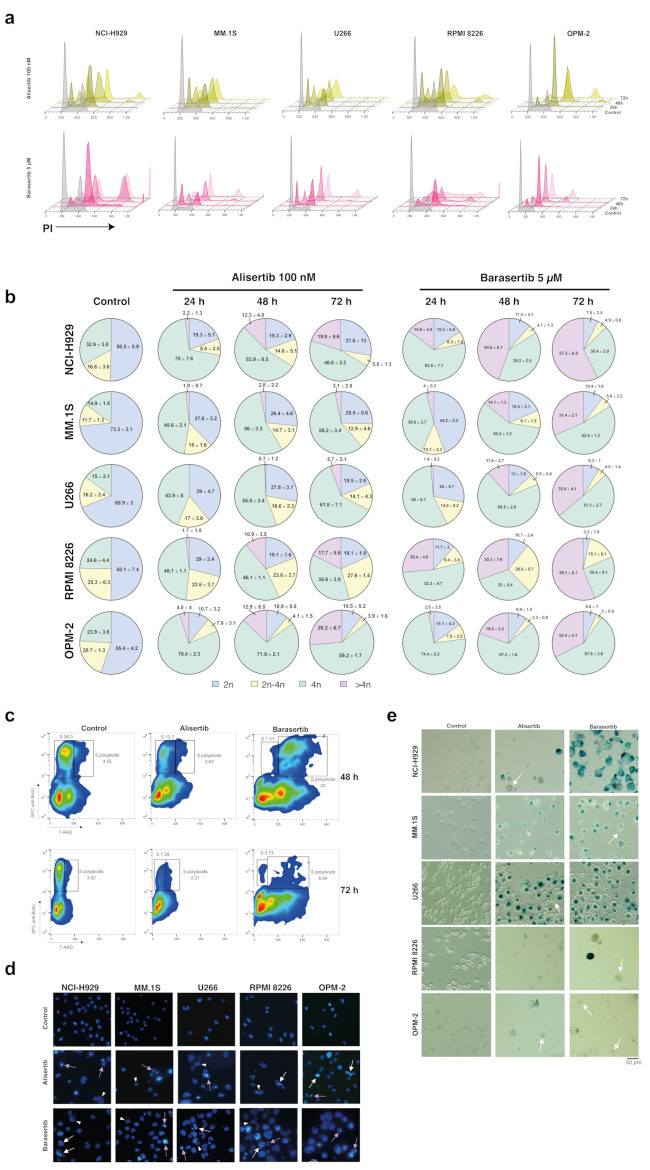
Aurora kinase inhibitors induce mitotic arrest, mitotic catastrophe and senescence in MM cell lines. MM cells were incubated with 100 nM alisertib (n = 3–7) and 5 µM barasertib (n = 3–6) for 24–72 h and DNA content was evaluated by flow cytometry. (**a**) Representative images of DNA content analysis of each cell line after treatments. (**b**) Distribution of cell populations with different DNA content for each drug and evaluated time. The global mean and SD are indicated for each phase. (**c**) Representative density plots of BrdU incorporation analysis in RPMI 8226 cells after treatment with 100 nM alisertib and 5 µM barasertib for 48 and 72 h (n = 2). (**d**) Nuclear morphology of MM cell lines treated with Aurora A and B inhibitors. Cells were incubated with 100 nM alisertib and 5 µM barasertib for 72 h and nuclei were stained with Hoechst 33342. Representative images of at least two independent experiments. White arrows show cells undergoing aberrant mitosis, white triangles refer to macro- and multinucleated cells, violet arrows indicate apoptotic cells and brown triangle points to micronucleated cells. (**e**) Senescence-associated β-galactosidase (SA β-gal) activity in MM cell lines treated with Aurora kinase inhibitors. MM cells were incubated with 5 µM alisertib or barasertib for 72 h, cultured for 5 days in the absence of the drugs and then stained with X-Gal. Representative images of at least two independent experiments. White arrows show dead cells.

Since Aurora A and Aurora B are essential mitosis regulatory proteins, the effect of alisertib and barasertib on the cell cycle of the aforementioned panel of MM cells was determined next. DNA content analysis by flow cytometry showed that alisertib (100 nM) and barasertib (5 µM) distorted the usual cell cycle profile of exponentially growing cells, causing prominent cell cycle arrest in the G2/M phase (4n) even after 24 h of treatment in all five cell lines tested (Figure 2a,b). While mitotic arrest remained after 72 h of incubation with alisertib, cells treated with barasertib during this period were more likely to undergo mitotic slippage and become polyploid. Nevertheless, alisertib also promoted the appearance of polyploid cells over time but to a lesser extent than barasertib. The DNA content of polyploid cells generated by alisertib treatment increased up to 8n, but reached greater than 8n in the case of barasertib (Figure 2a). Moreover, kinetics in the perturbation of cell cycle profiles varied among cell lines. On one hand, the greatest accumulation of NCI-H929, RPMI 8226 and OPM-2 cells with a 4n DNA content occurred 24 h after starting treatment (Figure 2b). However, MM.1S and U266 cells reached the 4n peak at 48 h with barasertib and 72 h with alisertib, probably due to the fact that the proliferative fraction in these cell lines was smaller. Strikingly, we detected in RPMI 8226 cells treated with barasertib for 48h a significant percentage of polyploid cells in the active S phase, as revealed by the BrdU assay (Figure 2c), suggesting endoreduplication. However, this population disappeared at 72 h due to cell death. These polyploid dead cells displayed a 2n-4n DNA content in cell cycle analysis (Figure 2a,b).

Mitotic arrest and defects in mitosis arising from the inhibition of Aurora proteins are associated with genomic instability [65]. In order to prevent this, higher eukaryotic cells can activate an oncosuppressive mechanism known as ‘mitotic catastrophe’ [66]. As shown in Figure 2d, alisertib and barasertib provoked aberrant mitosis with chromosome fragmentation (white arrows), and it was also possible to identify macro- and multinucleated giant cells (white triangles) as well as apoptotic cells (violet arrows).

To assess whether Aurora A and B inhibitors induced senescence in myeloma cells, measurement of senescence-associated β-galactosidase (SA β-gal) activity was performed. The results shown in Figure 2e confirm that both inhibitors induced entry into senescence. Nevertheless, the faint blue staining observed in RPMI 8226 and OPM-2 cells suggests a lower senescence-inducing potential of the drugs in these cell lines compared to others, possibly due to their higher sensitivity. As expected, dead cells (white arrows) were also detected across all cell lines.

### 3.3. Aurora Kinase Inhibitors Induce Changes in the Expression of Cell Cycle Regulator Proteins

**Figure 3:**
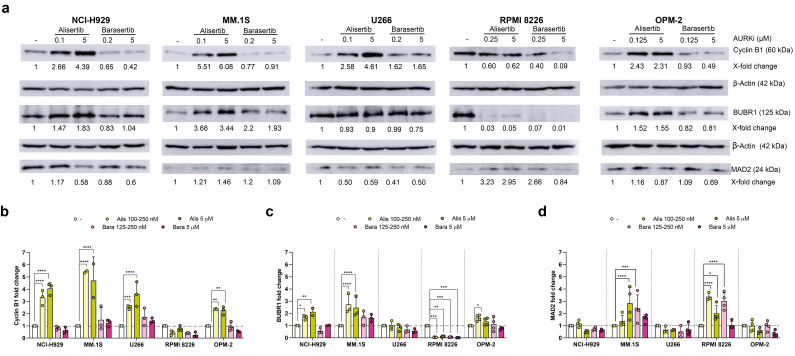
The expression of mitotic regulatory proteins in MM cells exposed to Aurora A and B inhibitors. Each cell line was treated with the indicated concentrations of alisertib and barasertib for 72 h and cell extracts were obtained. (**a**) Representative Western blot results from at least two independent experiments. Numbers indicate protein expression levels relative to control conditions and normalized to β-actin. (**b**–**d**) Quantitative analysis of changes in protein expression relative to controls. The global mean and SD are shown. Statistical analysis was performed using a two-way ANOVA test with Dunnet’s post-test, comparing the quantification of each protein for each treatment with respect to its baseline state. * *p* < 0.05; ** *p* < 0.01; *** *p* < 0.001; **** *p* < 0.0001. Original western blots are presented in File S1.

To further characterize the mitotic arrest induced by alisertib and barasertib in MM cells, the expression levels of cyclin B1, a protein that regulates the G2/M transition and mitotic exit [67], were determined. In most cases, we observed a significant increase in cyclin B1 expression after 72 h of treatment with alisertib, but not with barasertib. (Figure 3a,b). In addition, the expression of SAC components BUBR1 and MAD2 was also evaluated. As shown in Figure 3a,c, alisertib prompted an increase in BUBR1 levels in NCI-H929, MM.1S and OPM-2 cells, but not in U266 and RPMI 8226 cells. In fact, BUBR1 expression was almost completely abolished in RPMI 8226 cells after treatment with alisertib. In contrast, subtle changes in the levels of this protein were generally detected when cells were subjected to barasertib, except for RPMI 8226. Finally, the effect of Aurora A and B inhibitors on MAD2 expression levels was more heterogeneous. The most notable changes included a general increase in expression in MM.1S and RPMI 8226 cells, and a marked reduction in OPM-2 cells treated with the highest dose of barasertib (Figure 3a,d).

### 3.4. Aurora Kinase Inhibitor-Induced Cell Death Through Caspase-Dependent and Independent Mechanisms

**Figure 4:**
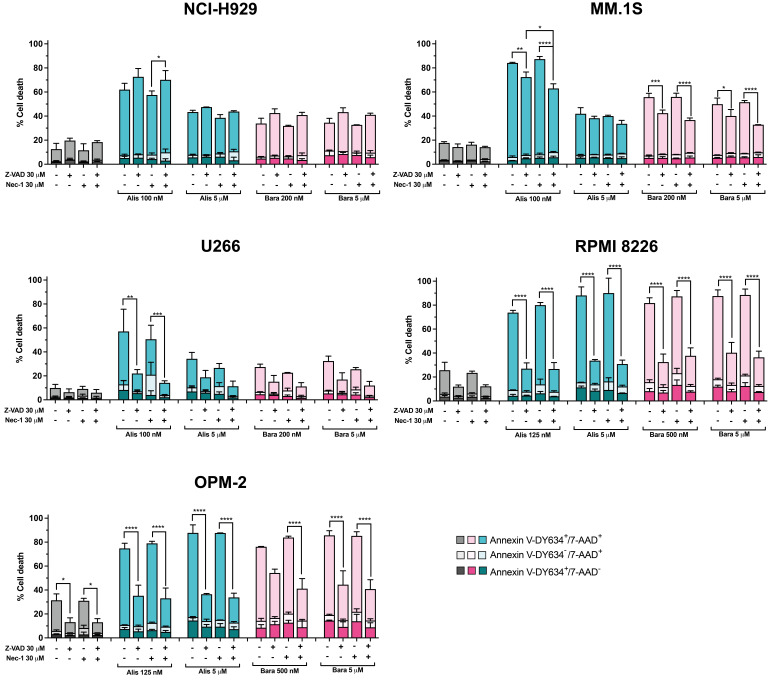
The involvement of caspases and RIPK1-mediated necroptosis in MM cell death triggered by Aurora kinase inhibitors. MM cells were treated with alisertib and barasertib at the indicated concentrations for 72 h in the presence/absence of the general caspase inhibitor Z-VAD and the RIPK1 inhibitor necrostatin-1. Cell death was assessed by Annexin V-DY634 binding/7-AAD staining and flow cytometry (n = 2–3), and the global mean and SD are represented. Two-way ANOVA with Tukey’s post-test was performed, comparing for each treatment the untreated cells with those treated with Z-VAD, and the latter and cells exposed to necrostatin-1 with those treated with Z-VAD+necrostatin-1. * *p* < 0.05; ** *p* < 0.01; *** *p* < 0.001; **** *p* < 0.0001.

Next, we evaluated the role of caspases and necroptosis in cell death induced by Aurora kinase inhibitors in MM cells. Pre-incubation with Z-VAD considerably reduced the cytotoxic activity of both compounds in U266, RPMI 8226 and OPM-2 cell lines (Figure 4). However, only a small protective effect was observed in MM.1S cells. In NCI-H929 cells, Z-VAD slightly sensitized them to these same treatments. We also used the RIPK1 inhibitor necrostatin-1 to determine whether RIPK1-mediated necroptosis, a type of caspase-independent cell death, was activated as a result of treatment with Aurora A and B inhibitors. The results indicate that pre-incubation with necrostatin-1 did not diminish the toxicity of the drugs in any cell line, except in U266 cells, although the difference was not statistically significant. Nevertheless, its combination with Z-VAD was equally or mildly more effective than Z-VAD alone in reducing the cytotoxic effect induced by alisertib or barasertib in MM.1S, U266 and OPM-2 cells.

### 3.5. Aurora Kinase Inhibitors Induce Changes in Mitochondrial Membrane Potential, as Well as Increases in Mitochondrial Mass and Mitochondrial ROS in MM Cells

**Figure 5:**
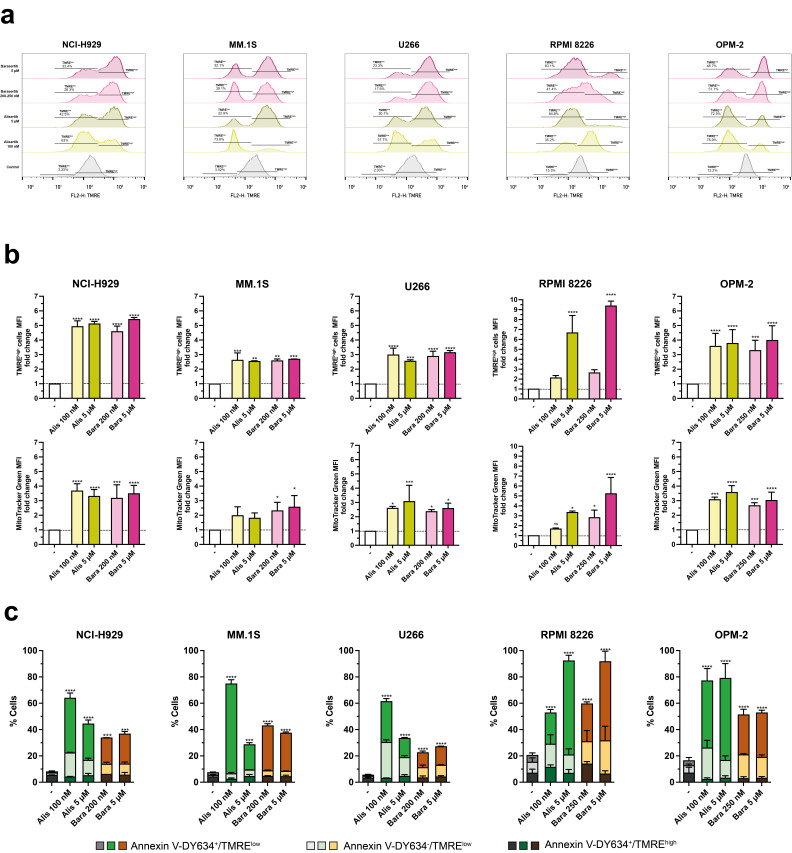
Changes in the mitochondrial transmembrane potential of MM cells after treatment with Aurora kinase inhibitors. (**a**) Representative histograms indicating the percentage of cells that lost mitochondrial membrane potential with each treatment. MM cells were incubated with alisertib and barasertib at the indicated concentrations for 72 h, and mitochondrial potential was assessed by labeling with the TMRE probe and flow cytometry. (**b**) A graphical representation of TMRE MFI changes in TMRE^high^ cells (n = 2–6) and the MFI of MitoTracker^TM^ Green FM (n = 2–5) induced by alisertib and barasertib respect to untreated cells (-). The global mean and SD are shown. Statistical analysis was performed using one-way ANOVA with Dunnett’s post-test, comparing each drug concentration to controls. * *p* < 0.05; ** *p* < 0.01; *** *p* < 0.001; **** *p* < 0.0001; ns, non significant. (**c**) Simultaneous determination of mitochondrial potential dissipation and induced cell death (n = 2–5) in control (-) or treated cells. Apoptosis was determined by Annexin V-DY634 binding. The global mean and SD are illustrated. One-way ANOVA with Dunnett’s post-test statistical analysis was performed, comparing each drug concentration to controls. *** *p* < 0.001; **** *p* < 0.0001.

Dissipation of mitochondrial transmembrane potential (ΔΨ_m_) is a key event in the intrinsic pathway of apoptosis [68]. Thus, we used the mitochondrial probe TMRE to study the effect of alisertib and barasertib in myeloma cells. The histograms corresponding to treated cells display two peaks (TMRE^high^ and TMRE^low^) (Figure 5a). We observed a global shift in the histograms in treated cells (Figure 5a) that could be due to hyperpolarization of mitochondria or to an increase in mitochondrial mass, related to the bigger size of the cells (Figure 2e). MFI of the TMRE^high^ population was 3–9 fold greater than that of control cells, depending on the cell line and inhibitor (Figure 5b, upper panels), with the greatest changes produced in NCI-H929 and RPMI 8226 cells. MitoTracker^TM^ Green FM staining demonstrated that alisertib and barasertib also provoked an increase in mitochondrial mass (Figure 5b, lower panels), but in RPMI 8226 and NCI-H929 cells the values derived from the analysis of mitochondrial potential were higher than those obtained from the analysis of mitochondrial mass, suggesting hyperpolarization of mitochondria. On the other hand, both inhibitors caused mitochondrial depolarization. Alisertib caused a greater mitochondrial potential collapse when used at low doses than at high doses in NCI-H929, MM.1S and U266 cells, according to the higher cytotoxicity observed at these concentrations (Figure 1). Likewise, a comparable level of mitochondrial potential dissipation was detected after treatment with both doses of barasertib in all cell lines except in RPMI 8226 cells, where the drug at the micromolar range had a greater effect than at doses in the nanomolar range (Figure 5a). Simultaneous analysis of mitochondrial potential and PS exposure was also performed (Figure 5c and Figure S1). Most of the TMRE^low^ cells were AnnexinV-DY634-positive in all cell lines, although a significant percentage of cells that lost ΔΨ_m_ were negative for Annexin V, indicating that loss of mitochondrial potential is an early event that precedes caspase activation and PS exposure. Moreover, in the case of RPMI 8226 cells treated with low doses of the inhibitors, it was also possible to identify cells that preserved mitochondrial potential but exposed PS on their surface (Figure 5c and Figure S1).

### 3.6. Alisertib Activates Bax/Bak-Independent Cell Death Mechanisms Involving Cytochrome c Release in MM Cells

**Figure 6:**
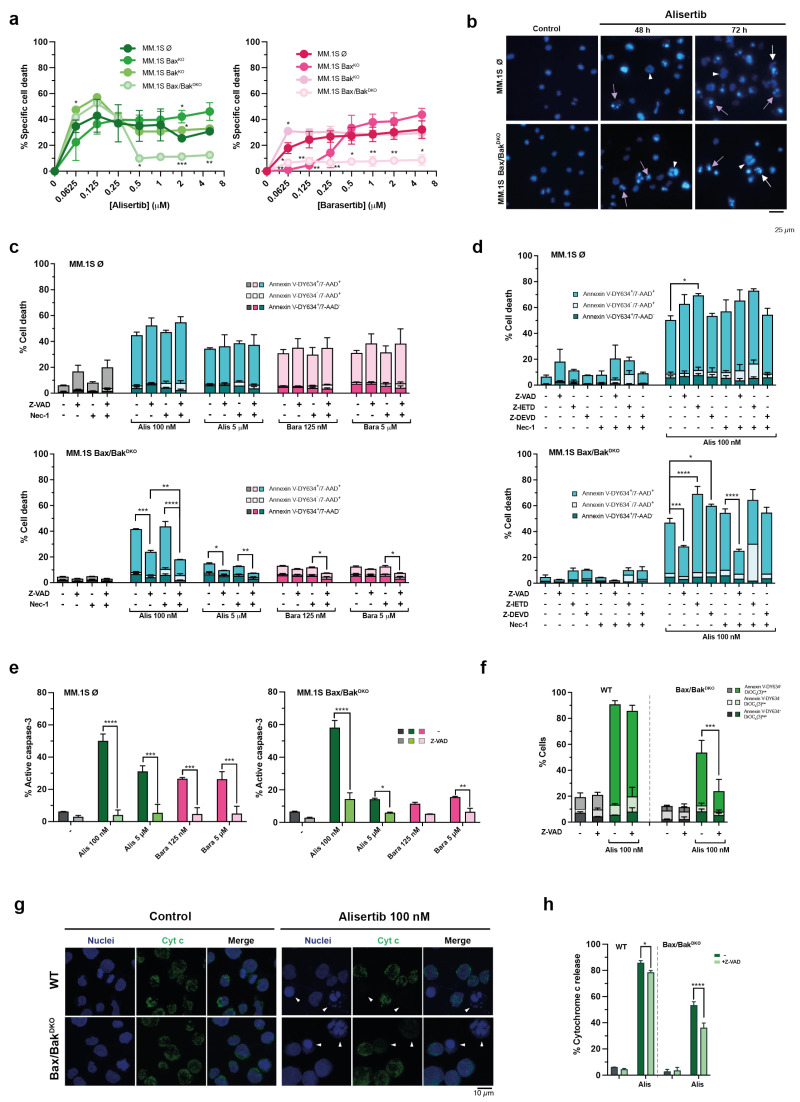
The sensitivity of MM.1S Bax^KO^, MM.1S Bak^KO^ and MM.1S Bax/Bak^DKO^ cells to Aurora kinase inhibitors. (**a**) CRISPR-Cas9-modified MM.1S cells were treated for 72 h with increasing concentrations of alisertib and barasertib (0–5 µM), and cell death was determined by Annexin V-DY634 binding/7-AAD staining and flow cytometry (n = 2–4). Specific cell death is depicted and the global mean and SD are illustrated. Statistical analysis was performed by using a two-tailed unpaired t-test, comparing each drug concentration in the Bax^KO^, Bak^KO^ and Bax/Bak^DKO^ cells to the infected control line. * *p* < 0.05; **p < 0.01; *** *p* < 0.001. (**b**) Alterations in the nuclear morphology of MM.1S control and DKO cells lines treated with alisertib for 48 h or 72 h. After treatment, cells were stained with Hoechst 33342. Representative images of at least two independent experiments. White arrows show cells undergoing aberrant mitosis, white triangles refer to macro- and multinucleated cells and violet arrows indicate apoptotic cells. (**c**,**d**) The involvement of caspases and RIPK1-mediated necroptosis in cell death triggered by Aurora kinase inhibitors in MM.1S Bax/Bak^DKO^ cells. Cells were treated with alisertib and barasertib at the indicated concentrations for 72 h in the presence/absence of the general caspase inhibitor Z-VAD or the specific inhibitors Z-IETD and Z-DEVD and the RIPK1 inhibitor necrostatin-1. Cell death was assessed by Annexin V-DY634 binding/7-AAD staining and flow cytometry (n = 2–4), and the global mean and SD are represented. Two-way ANOVA with Tukey’s post-test was performed, comparing for each treatment the untreated cells with those treated with each caspase inhibitor and both the latter group and those exposed to necrostatin-1 with those treated with Z-VAD+necrostatin-1. * *p* < 0.05; ** *p* < 0.01; *** *p* < 0.001; **** *p* < 0.0001. (**e**) Caspase-3 activation in MM.1S and MM.1S Bax/Bak^DKO^ cells. Cells were exposed to the indicated doses of alisertib and barasertib for 72 h in the presence/absence of Z-VAD, and the level of caspase-3 activation was determined. Data from at least two independent experiments and the global mean and SD are shown. Statistical analysis was performed using two-way ANOVA with Sidak’s or Dunnett’s post-test, comparing each drug concentration in the presence or absence of Z-VAD. * *p* < 0.05; ** *p* < 0.01; *** *p* < 0.001; **** *p* < 0.0001. (**f**) Simultaneous assessment of mitochondrial potential disruption and induced cell death ¡(n = 2–3) by labeling with the mitochondrial probe DiOC_6_(3) and Annexin V-DY634 binding, respectively, as well as flow cytometry. The global mean and SD are shown. Two-way ANOVA with Sidak’s post-test was performed, comparing the effect of the drug in the presence and absence of Z-VAD. *** *p* < 0.001. (**g**) Confocal immunofluorescence images of cytochrome c localization. After 48 h of incubation with 100 nM alisertib, cells were fixed and immunostained with an anti-cytochrome c antibody (clone 6H2.B4) and Alexa 488-conjugated anti-mouse IgG antibody. Nuclei were stained with Hoechst 33342. Representative images from at least two independent experiments. White triangles point to cells that have released cytochrome c from mitochondria. (**h**) A graphical representation of cytochrome c efflux after treatment in each cell line (n = 2–3). The global mean and SD are represented. Two-way ANOVA with Sidak’s post-test was performed, comparing the effect of the drug in the presence and absence of Z-VAD. * *p* < 0.05; **** *p* < 0.0001.

To assess whether the mitochondrial apoptotic pathway is involved in the effect of Aurora kinase inhibitors in MM cells, we assessed the sensitivity of single or combined Bax and Bak deletion mutants of MM.1S cells to Aurora A and B inhibitors. The results indicate that barasertib-driven death occurs mainly through the mitochondrial apoptotic pathway, and it is more dependent on Bax than on Bak at the lowest doses tested (62.5–250 nM) (Figure 6a). Interestingly, the intrinsic apoptotic pathway was also the main death mechanism activated by alisertib at high concentrations (≥500 nM), but at lower doses other alternative cell death mechanisms independent of Bax/Bak were activated (Figure 6a). This dual response of MM.1S Bax/Bak^DKO^ cells to alisertib was also detected after shorter treatments (Figure S2). In fact, apoptotic nuclei were also found in these cells when exposed to low-dose alisertib (Figure 6b). On the other hand, no clear dependence on Bax or Bak was identified in cell death triggered by alisertib (Figure 6a).

As illustrated in Figure 6c, pre-incubation of Z-VAD accompanied or not by necrostatin-1 partially prevented cell death induced by alisertib and barasertib in MM.1S Bax/Bak^DKO^ cells, especially that induced by alisertib at low doses, but not in MM.1S Ø control cells. Furthermore, the protection achieved in these cells against alisertib was greater than that observed in the parental cell line (Figure 4). Specific inhibition of caspase-8 did not protect Bax/Bak^DKO^ cells from alisertib-induced death, but we observed an increase in the necrotic population in MM.1S Bax/Bak^DKO^ cells treated with alisertib in the presence of Z-IETD and necrostatin-1 (Figure 6d). Again, necrostatin-1 alone was not able to reduce the toxicity of the compounds in any case. We confirmed caspase-3 activation and its inhibition by Z-VAD (Figure 6e). In the presence of Z-VAD, caspase-3 activity decreased near basal levels with all treatments in the modified cell lines and also in MM.1S control cells treated with barasertib and 5 µM alisertib. Interestingly, this occurred even when cells death was not inhibited or was only partially inhibited (Figure 6c,d).

MOMP involves loss of ΔΨ_m_ and the release of cytochrome c from mitochondria, which are central events in apoptotic signaling in the intrinsic pathway, triggering the caspase activation cascade and also caspase-independent cell death. Since MM.1S Bax^/^Bak^DKO^ cells die in response to alisertib doses in the nanomolar range and caspase-3 is activated in these cells, we decided to determine whether mitochondrial transmembrane potential was dissipated and cytochrome c reached the cytosol in this context through a Bax/Bak-independent mechanism. Analysis of mitochondrial transmembrane potential and Annexin V binding showed that alisertib induced mitochondrial depolarization in Bax/Bak^DKO^, but in contrast with Bax/Bak-proficient cells, caspase inhibition significantly prevented the loss of ΔΨ_m_ (Figure 6f). Moreover, the percentage of cells that lost ΔΨ_m_ but were still negative for Annexin V-DY634 was smaller in Bax/Bak^DKO^, suggesting that mitochondrial depolarization could be dependent on caspase activity in these cells. Cytochrome c release was assessed through immunofluorescence and flow cytometry (Figure 6g,h and Figure S3). Histograms in Figure S3 show a rightward shift of the peak corresponding to the cell population retaining cytochrome c in the mitochondria. This shift is likely due to an increase in cell size and the number of mitochondria per cell as a consequence of the drug’s action. Unsurprisingly, cytochrome c was released in the MM.1S wild-type treated with alisertib, but Z-VAD failed to effectively prevent cytochrome c release (Figure 6h). Interestingly, our data suggest that cytochrome c could also reach the cytosol in MM.1S Bax/Bak^DKO^ cells under the same treatment conditions, since two overlapping peaks are observed, and Z-VAD would partially block such release (Figure 6f and Figure S3). This effect on ΔΨ_m_ loss and cytochrome c release observed with Z-VAD partially fits with a caspase-mediated amplification loop on mitochondria, previously described by other authors [69,70].

### 3.7. Pharmacological Inhibition of Aurora A and B Enhances Mitochondrial ROS Production in MM Cells

**Figure 7:**
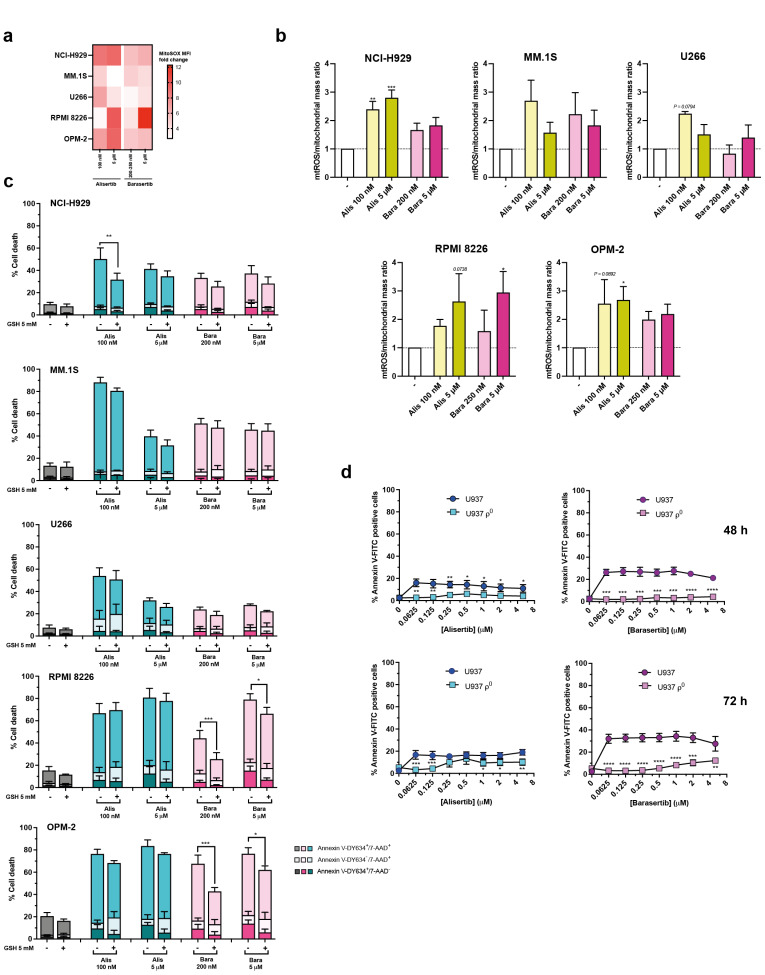
ROS production and its role in MM cell death induced by Aurora kinase inhibitors. (**a**) MM cells were exposed to the indicated doses of alisertib and barasertib for 72 h and mitochondrial ROS (mtROS) production was assessed using the mitochondrial probe MitoSOX^TM^ and flow cytometry. MitoSOX MFI increase (mean from 2–5 independent experiments) is represented for each cell line and treatment. (**b**) mtROS production relative to control cells (-) normalized to mitochondrial mass (MitoSOX/ MitoTracker^TM^ Green FM MFI ratio). The data are from 2–5 independent experiments. The global mean and SD are shown. Statistical analysis was performed using one-way ANOVA with Dunnett’s post-test, comparing each treatment to the control. * *p* < 0.05; ** *p* < 0.01; *** *p* < 0.001; (**c**) The effect of inhibition of total ROS production in MM cells exposed to alisertib and barasertib. Cells were treated with drugs at the indicated doses in the presence/absence of the antioxidant glutathione (GSH), and triggered cell death was determined by Annexin V-DY634 binding/7-AAD staining and flow cytometry (n = 3–7). The global mean and SD are represented. Two-way ANOVA with Sidak’s post-test was performed, comparing the effect of each drug concentration in the presence and absence of GSH. * *p* < 0.05; ** *p* < 0.01; *** *p* < 0.001. (**d**) The sensitivity of U937 ρ^0^ cells to Aurora A and B inhibitors. U937 wild-type and ρ^0^ cells were treated for 48–72 h with increasing concentrations of alisertib and barasertib (0–5 µM) and induced apoptosis was determined by Annexin V-FITC binding and flow cytometry (n = 3–4). The global mean and SD are illustrated. Statistical analysis was performed using the two-tailed unpaired t-test statistical analysis, comparing each drug concentration in U937 ρ^0^ cells to wild-type cells. * *p* < 0.05; ** *p* < 0.01; *** *p* < 0.001; **** *p* < 0.0001.

To delve into the damage caused by alisertib and barasertib in mitochondria, we next studied the effect of both inhibitors on the specific generation of mitochondrial ROS (mtROS). MitoSOX MFI, relative to control cells (Figure 7a), suggests that the two inhibitors enlarged ROS production in mitochondria in all cell lines after 72 h of drug action, with variations according to cell lines, compounds and the dose used. However, the increase in cell size and mitochondrial mass promoted by the inhibitors contributed to massive mitochondrial ROS production. Therefore, the mtROS/mitochondrial mass ratio, relative to the amount of ROS produced by each mitochondrion, was determined, and the data obtained denoted that this ratio also increased in all five cell lines, although this increase was statistically significant only in NCI-H929 and OPM-2 cells treated with alisertib and RPMI 8226 cells treated with barasertib (Figure 7b).

Furthermore, we also evaluated whether total ROS were involved in the toxicity induced by alisertib and barasertib in MM cells, using the antioxidant glutathione (GSH). According to our results, GSH partially protects against the cytotoxic activity of 100 nM alisertib in NCI-H929 and barasertib in RPMI 8226 and OPM-2 cells, especially at low doses, after 72 h of treatment (Figure 7c). Finally, the response to Aurora kinase inhibitors was evaluated in human pro-monocytic leukemia U937 cells lacking mitochondrial DNA (U937 ρ^0^). Morphologically, the inhibitors also induced an increase in cell size in both U937 wild-type and ρ^0^ cells (Figure S4). Furthermore, prolonged exposure to the compounds promoted apoptosis (purple arrows), especially in the parental cell line, and necrotic cells (blue arrows) could also be identified (Figure S4). Analysis of Annexin V binding revealed that U937 ρ^0^ cells were highly resistant to the cytotoxic effect of alisertib and, in particular, barasertib even after 72 h of incubation compared to wild-type cells (Figure 7d).

### 3.8. BH3-Mimetics and Panobinostat Synergize with Aurora Kinase Inhibitors in MM Cell Lines

In an attempt to improve the efficacy of Aurora A and B inhibitors, we tested their combination with other antitumor agents. To do this, we adjusted single drug concentrations for each cell line so that the level of death induced did not exceed 30–40%. We used the experimentally observed (OBS) specific apoptosis and calculated the hypothetical expected (EXP) specific apoptosis, previously used by Nijhof and collegues [64]. EXP assumes an additive effect of the two combined drugs, and it was calculated using the following formula: [(% apoptosis Aurora kinase inhibitor + % apoptosis drug X) − (% apoptosis Aurora kinase inhibitor x % apoptosis drug X)/100]. This was previously used by Cuenca and co-workers [71]. Finally, the difference OBS-EXP was calculated to categorize a combination as synergistic, additive or antagonistic.

**Figure 8:**
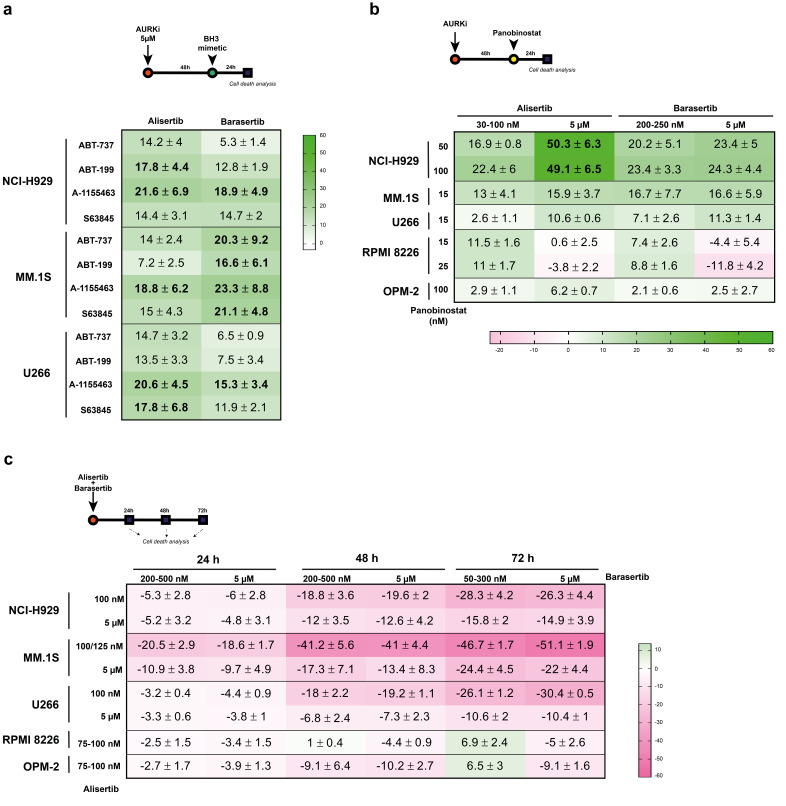
The combination of Aurora kinase inhibitors with different antitumor agents in MM cell lines. (**a**) MM cells were treated with 5 µM alisertib and barasertib for 48 h, followed by the addition of BH3-mimetics at the indicated concentrations, incubating the combination for an additional 24 h (n = 2–5). (**b**) MM cells were treated with alisertib and barasertib at the indicated doses for 48 h. Panobinostat was then added at the indicated concentrations, and the combination was incubated for another 24 h (n = 3–5). (**c**) MM cells were simultaneously incubated with alisertib and barasertib at the indicated concentrations for 24–72 h (n = 3–7). In all cases, triggered cell death was assessed by Annexin V-DY634 binding/7-AAD staining and flow cytometry. The toxicity of each single drug concentration was adjusted to a maximum of 30–40%. The combinations were considered synergistic when [empirically observed specific cell death (OBS) − hypothetical expected specific cell death (EXP)] > 10 units. Green indicates synergism and pink indicates antagonism. The global mean and SD are shown.

Given the strong dependence of MM cells on anti-apoptotic proteins of the Bcl-2 family [72], we first evaluated the combination of Aurora kinase inhibitors with BH3-mimetics. Since alisertib and barasertib induce senescence in myeloma cells, we sought to evaluate the feasibility of a “one-two punch strategy” [73] for myeloma, using BH3-mimetics as potential senolytics. In particular, the Bcl-2 inhibitor (ABT-199), Bcl-X_L_ inhibitor (A-1155463), Mcl-1 inhibitor (S63845) and Bcl-2, Bcl-X_L_ and Bcl-w inhibitor (ABT-737) were used. Specifically, cells were exposed to 5 µM alisertib/barasertib for 48 h, and then BH3-mimetics were added, leaving the combination to act for another 24 h. This drug administration scheme led to modest synergies, as seen in Figure 8a and Figure S5. In general, the combination of BH3-mimetics with alisertib was slightly more beneficial than with barasertib in NCI-H929 and U266 cells, though the combination of S63845 with either alisertib or barasertib in NCI-H929 cells induced a similar synergism. However, BH3-mimetics strengthened the cytotoxic activity of barasertib to a greater extent compared to alisertib in MM.1S cells. Even so, A-1155463 was the most successful BH3-mimetic in enhancing apoptosis in all three cell lines, although ABT-737, S63845 and A-1155463 equivalently sensitized MM.1S cells to the action of barasertib.

Sequential combination of Aurora kinase inhibitors with drugs approved for clinical use in MM was also tested. This was the case with the oral pan-histone deacetylase inhibitor panobinostat [74] that has been shown to enhance the activity of Aurora kinase inhibitors in T-cell lymphoma [75] and prostate cancer [76]. On this occasion, cells were treated for 48 h with high and low doses of alisertib and barasertib; panobinostat was then added, and the combination was incubated for an additional 24 h. Interestingly, our results reveal a robust synergistic effect in NCI-H929 cells only when panobinostat was combined with 5 µM alisertib (Figure 8b and Figure S5b). The efficacy of the combinations decreased in MM.1S cells, especially in U266, RPMI 8226 and OPM-2 cells, where poor synergism, an additive and even an antagonistic effect were observed.

Finally, we tested the co-incubation of alisertib and barasertib to assess whether targeting mitosis at different levels could induce a synergistic effect. Figure 8c and Figure S5c reveal a marked time-dependent antagonistic effect in NCI-H929, U266 and, especially, MM.1S cell lines. Furthermore, this antagonism was more potent when barasertib was combined with low doses of alisertib. However, additivity or a milder antagonistic effect occurred in RPMI 8226 and OPM-2 cells.

## 4. Discussion

The incorporation of targeted therapies and immunotherapy into the therapeutic arsenal for multiple myeloma (MM) in recent decades has significantly prolonged patient survival. However, many patients eventually relapse and become refractory, often as a result of developing drug resistance [3]. Therefore, this scenario calls for the urgent need to find effective alternatives, such as novel targeted agents and more potent drug combinations. In this regard, Aurora kinase inhibitors are potential candidates to fight MM, since Aurora A overexpression is relatively frequent in myeloma cells [77]. Although numerous preclinical studies have demonstrated the efficacy of these drugs, the clinical data are generally discouraging [78]. Yet alisertib, an Aurora A antagonist, is the first-in-class inhibitor that has shown modest anti-myeloma activity alone and in combination with bortezomib in clinical trials [36,79]. Here, we have investigated the mechanism of action of alisertib and barasertib, an Aurora B inhibitor with reported anti-myeloma activity [80], and explored their combination with novel antitumor agents in vitro. We found that both drugs reduced cell proliferative capacity and induced apoptosis to a greater extent when treatments were prolonged, which was consistent with previous studies [58,80]. In addition, we also observed that alisertib showed more potent antitumor activity than barasertib. Interestingly, the dual dose-dependent response of NCI-H929, MM.1S and U266 cells to alisertib suggests that this drug displays two different mechanisms of action in these cell lines. Since the expression levels of Aurora A and Aurora B are similar across all the cell lines tested, this differential behavior is likely due to intrinsic cellular factors rather than differences in kinase expression. In line with this, Hose et al. reported that sensitivity to the pan-Aurora inhibitor VX-680 was not associated with Aurora A/B expression levels, based on a study involving 20 myeloma cell lines and five ex vivo myeloma cell samples [17].

Although alisertib is known as a highly specific Aurora A inhibitor, several studies point to the ability of the drug to inhibit Aurora A and B differentially according to the dose, with Aurora A being inhibited at low doses, and Aurora B also at high concentrations [81]. We align our results with this hypothesis, since the antitumor effect of alisertib is greater at low doses than at high doses, and in the latter case, the effect of Aurora B blockade would predominate.

Cell cycle arrest in the G2/M phase is a common event associated with the blockage of mitotic regulatory proteins, including Aurora proteins [37,82]. As expected, we confirmed that low-dose alisertib and high-dose barasertib induced mitotic arrest in all five cell lines. However, the cell cycle progression associated with each treatment and the expression levels of cyclin B1 indicate that both drugs induce mitotic arrest through different mechanisms. It is well-known that the cyclin B1/CDK1 complex is essential for proper G2/M transition, and its activity is highly dependent on cyclin B1 expression, which peaks at the G2/M transition and declines at the onset of anaphase [83]. The relatively low levels of cyclin B1 after prolonged exposure to barasertib are consistent with a transient mitotic arrest that cells avoid through mitotic slippage, since inhibition of Aurora B involves the abolition of the spindle assembly checkpoint (SAC), and consequently, cyclin B1 is rapidly degraded. In contrast, the accumulation of cyclin B1 after treatment with alisertib would correlate with a prolonged mitotic arrest, since failures in mitotic spindle assembly caused by Aurora A inhibition would keep the SAC activated and prevent cyclin B1 degradation. The exception occurs in RPMI 8226 cells, since low levels of cyclin B1 and the high percentage of polyploid cells after alisertib treatment could indicate that cells exit mitotic arrest. Several studies have detected a decrease in cyclin B1 levels and an increase in cell cycle progression inhibitors p21 and p27 upon alisertib-induced cell cycle arrest in breast, ovarian and pancreatic cancer cells [37,38,84]. Moreover, another study found that up to 250 nM alisertib, cyclin B1 expression increased, but at higher concentrations cyclin B1 levels decreased due to a shift towards Aurora B inhibition, whose phenotype was dominant in HeLa cells [85]. Interestingly, we agree that alisertib in the micromolar range induces an effect consistent with Aurora B inhibition, but we observed elevated cyclin B1 expression in NCI-H929, MM.1S, U266 and OPM-2 cells. Therefore, this could indicate that under these conditions Aurora B inhibition prevents cell death, although Aurora A is inhibited.

Mitotic arrest and defects in mitosis arising from antimitotic drugs, including Aurora kinase inhibitors, are associated with genomic instability [65]. To prevent this, cells may activate an oncosuppressive mechanism known as ‘mitotic catastrophe’ [66], which is morphologically characterized by unique nuclear alterations including macro-, multi- and micronucleation [86]. The observation of these nuclear peculiarities corroborates that MM cells activate mitotic catastrophe in response to low doses of alisertib and high doses of barasertib. Once this mechanism is activated, the possible cell outcomes are death, aneuploidy, entry into a senescent state or the induction of polyploidy after mitotic slippage [66]. Taken together, our results reveal that both alisertib and barasertib lead to cell death, entry into senescence and the induction of polyploidy through one or several cycles of endoreduplication in all MM cell lines, even when cyclin B1 levels remain elevated. This is in line with several studies demonstrating the induction of senescence upon inhibition of Aurora proteins in numerous cellular and animal models [87,88,89]. Furthermore, our study supports others which have also reported the induction of polyploidy associated with Aurora A, even though Aurora A blockade is generally related to aneuploidy and Aurora B to polyploidy [90]. However, the role of p53 in the induction of polyploidy by inhibiting Aurora A is controversial, as some reports argue that wild-type p53 is associated with apoptosis induction and its absence or a mutated form with polyploidy induction [19], while others have observed that p53 status does not influence final cell fate [91]. In our case, since the population of polyploid and apoptotic cells increases in cells that have wild-type p53 (NCI-H929 and MM.1S cells) and in those that have mutated p53 (U266, RPMI 8226 and OPM-2) [92], we deduce that p53 is not involved in the choice of cell fate after the action of alisertib.

There is evidence that antimitotic drugs, including Aurora kinase inhibitors, trigger the intrinsic apoptotic pathway, which is thoroughly governed by Bcl-2 family proteins [93]. Bax and Bak are canonical effector proteins of the intrinsic apoptotic pathway [47]. Considering that Bok, the third effector protein of the family, low expressed in MM [94], and the results derived from dual-gene suppression of Bax and Bak, we conclude that the main cytotoxic mechanism triggered by alisertib at high doses and by barasertib is the mitochondrial apoptotic pathway, while alisertib at low doses activates an alternative Bax/Bak-independent cell death mechanism, in contrast with other reports on colorectal cancer [93] and Burkitt lymphoma [95]. In addition, in this last study, they also found that Bax was important in alisertib-induced cell death, while Sak et al. [96] deduced that Bak was important in alisertib toxicity in glioblastoma cells. However, the data derived from the single silencing of Bax/Bak do not allow us to draw firm conclusions about their involvement at an individual level in alisertib-triggered cell death in MM, although in the case of barasertib, Bax would be involved in cell death when used at low doses. This prominent role of Bax in Aurora B inhibition has been previously proposed [95], although other studies confer a determining role to Bax and Bak individually [93].

Bax/Bak-driven MOMP is considered the point of no return of the mitochondrial apoptotic pathway, which is accompanied by the dissipation of the mitochondrial membrane potential and the release of cytochrome c from the mitochondrial intermembrane space into the cytosol. Coinciding with our results, both events had been previously reported in different cellular models of solid and blood cancers by pharmacologically inhibiting Aurora proteins [84,97,98]. Moreover, we demonstrated that the mitochondrial potential is also lost and that cytochrome c is released from mitochondria even in the absence of effector proteins, so this would support the proposal of the existence of Bax/Bak-independent mechanisms that lead to mitochondrial potential disruption and allow for cytochrome c release [99,100]. Furthermore, we observed an apparent hyperpolarizing effect of the drugs on mitochondria, but the analysis of mitochondrial mass revealed that this effect only occurred in NCI-H929 cells and, especially, in RPMI 8226 cells. Since one of the causes of mitochondrial hyperpolarization in tumor cells is related to the ‘Warburg effect’ [101], this observation is consistent with the fact that RPMI 8226 is one of the fastest proliferating cell lines and possesses high glycolytic activity under basal conditions, as previously described [102,103]. We therefore speculate that RPMI 8226 cells enhance glycolysis in the presence of high doses of alisertib and barasertib, causing hyperpolarization of the inner mitochondrial membrane, but this would not occur when they are used at low doses. There are very few studies exploring the relationship between the inhibition of Aurora family proteins and cellular metabolism. Only Nguyen et al. have described metabolic reprogramming in glioblastoma cells using alisertib, managing to suppress glycolysis and activate oxidative phosphorylation [97], so it would be interesting to delve deeper into this field.

Several studies have proposed that cell death induced by Aurora kinase inhibitors, including alisertib and barasertib, is caspase-dependent [30,104,105], but our work suggests that the involvement of caspases in drug-induced cell death is cell line-dependent, and that RIPK1-mediated necroptosis is not activated in any case. Likewise, caspase-8 would not be involved in the toxicity induced by low-dose alisertib in Bax/Bak-deficient cells. However, the fact that Z-VAD partially protects them suggests the mediation of caspases other than -8 and -3 and the activation of other cell death mechanisms. In this regard, the activation of caspase-2 has recently been linked to the inhibition of Aurora proteins [106,107]. Additionally, it has been described that caspase-2 and -8 can trigger cell death through a Bax/Bak-independent mechanism in colon cancer cells treated with resveratrol [108]. Taking into account these reports and knowing that Z-VAD is a pan-caspase inhibitor that blocks caspase-1 to -10, except for caspase-2 [109], it is possible that in our case caspase-2 could be involved in cell death induced by alisertib and barasertib in some cell lines. However, other antimitotic drugs, like vincristine, have been shown to induce cell death independently of caspase-2 [110]. Furthermore, the increase in the necrotic population in cells lacking Bax and Bak when exposed to low doses of alisertib and caspase-8 and RIPK1 inhibitors could indicate that this treatment leads the cells to a necrotic morphotype cell death by some alternative mechanism. One of them could be pyroptosis, a type of programmed cell death mediated by inflammatory caspases and gasdermins [86], but further studies are needed to confirm or refute this hypothesis.

Pharmacological inhibition of Aurora proteins has been shown to increase ROS production, the effects of which are associated with the induction of autophagy, cell death, polyploidy and senescence [111,112]. Here, we deduce that total ROS partially contribute to the cytotoxic activity of alisertib and barasertib in certain cell lines, especially when used at low doses, since the antioxidant GSH partially prevented cell death induced by the drugs. This is in agreement with previous studies reporting at least partial neutralization of the effects associated with excessive ROS generation by different ROS scavengers [112,113,114]. We also found that both inhibitors enhanced ROS production in mitochondria (mtROS), which are the major source of ROS generation [115], and by using leukemic cells devoid of mitochondrial DNA, we suggest that mtROS participate in the cytotoxic potential of the drugs.

In recent decades, drug combinations have proven to be more effective than monotherapy in many cancer types, including MM. We therefore examined the efficacy of combining Aurora kinase inhibitors with different antitumoral agents under investigation or in clinical use in MM cell lines. Since alisertib and barasertib induce senescence in MM cells, we decided to evaluate the feasibility of a “one-two punch strategy” [73]. In this case, we used BH3-mimetics as potential senolytic agents for MM, since several studies have previously demonstrated their senolytic activity [116]. We found that the sequential combination of high-dose Aurora kinase inhibitors and multitarget or single-protein BH3-mimetics was modestly synergistic and more effective than when both families of drugs were incubated simultaneously. This administration scheme has already been shown to be effective when BH3-mimetics were added to other antitumor drugs in vitro and in vivo in different tumor cell models [117,118,119]. Our data suggest that Bcl-X_L_ inhibition could be an efficacious strategy to improve the toxicity of Aurora A and B inhibitors, but it is essential to avoid thrombocytopenia associated with the blockade of this anti-apoptotic protein [53]. We also found variable efficacy when sequentially combining alisertib or barasertib with the pan-histone deacetylase inhibitor panobinostat, resulting in robust synergism in NCI-H929 cells pretreated with high doses of alisertib. Previous studies have also demonstrated the efficacy of combining histone deacetylase inhibitors with standard MM treatments [120]. However, the selective synergism observed in this work requires further investigation into the molecular characteristics that make this combination highly synergistic in certain MM subtypes and whether it may be effective in any group of patients. Also, studying the effect of the microenvironment on the efficacy of these combinations would be interesting. In this sense, Görgün et al. have shown that BM stromal cells or cytokines do not hinder the anti-myeloma activity of Aurora kinase inhibitors [58]. Finally, we detected a potent antagonistic effect that was strengthened over time in three of the five cell lines when alisertib and barasertib were simultaneously combined. We hypothesize that this antagonism could be a consequence of the predominance of the inhibitory effect of Aurora B over that of Aurora A, especially when alisertib was used at low doses. Our hypothesis is supported by studies carried out with pan-Aurora inhibitors. For example, Reiman et al. observed that VE-465 induced polyploidy and cell death in MM cells in a manner similar to that of AZD1152 (barasertib) [121]. Likewise, in other studies with different cell types of lymphomas and solid tumors, it was found that endoreduplication associated with the action of AT9283, VX-680 and PHA-739358 (danusertib) reflected a phenotype similar to the inactivation of Aurora B [53,122,123,124,125].

## 5. Conclusions

The incurability of MM requires the search for new therapeutic approaches that allow to combat the disease more effectively. In this study, we have demonstrated that Aurora A (alisertib) and B (barasertib) inhibitors displayed anti-myeloma activity, inducing mitotic arrest leading to polyploidy, entry into senescence and cell death, with caspases and mtROS as potential contributors to drug toxicity. Both inhibitors triggered the mitochondrial apoptotic pathway, and alisertib also activated alternative cell death mechanisms involving the release of cytochrome c through a Bax/Bak-independent mechanism. In addition, when the novel agents BH3-mimetics and panobinostat were combined sequentially, they synergized in some cases, emerging as a promising strategy for MM in early-stage studies. However, the efficacy of Aurora kinase inhibitors and their combinations should be confirmed in more advanced phases of research to assess their potential clinical use. Furthermore, our results demonstrate that myeloma cells are heterogenous in the mechanisms activated by these drugs. Thus, more exhaustive studies are needed to identify potential markers that can help predict treatment response in order to find the best therapeutic option for each MM subtype.

## Data Availability

The original contributions presented in this study are included in the article. Further inquiries can be directed to the corresponding author.

## Supplementary Materials

The following supporting information can be downloaded at https://www.mdpi.com/article/10.3390/cancers17142290/s1: Supplementary Figure S1. Simultaneous analysis of mitochondrial transmembrane potential and PS exposure in MM cells treated with alisertib or barasertib. Cells were treated for 72 with the indicated concentrations of AURKi, stained with TMRE and AnnexinV-DY634 and analyzed by and flow cytometry. Representative dot-plots are shown. Supplementary Figure S2. Sensitivity of Bax/Bak^DKO^ MM.1S cells to alisertib. Genetically modified MM cells were treated for 24 and 48 hours with increasing concentrations of alisertib (0–1 μM) and cell death was assessed by Annexin V-DY634 binding/7-AAD staining and flow cytometry (n = 2–7). Specific cell death is depicted and global mean and SD are represented. Two-tailed unpaired t-test statistical analysis was performed, comparing each drug concentration in the Bim-deficient lines to the infected control line. * *p* < 0.05; ** *p* < 0.01. Supplementary Figure S3. Representative histograms from flow cytometry analysis indicating the percentage of cytochrome c release into the cytosol with each treatment in MM.1S and MM.1S Bax/Bak^DKO^ cells. Supplementary Figure S4. Morphological changes induced by Aurora kinase inhibitors in U937 ρ^0^ cells. U937 wild-type and ρ^0^ cells were treated for 72 h with increasing concentrations of alisertib and barasertib (0–5 μM). Purple arrows point to apoptotic cells, blue arrows indicate necrotic cells and black irregular shapes show spherical cell debris. Scale bar = 25 μm (U937 wild-type cells) and 50 μm (U937 ρ^0^ cells). Supplementary Figure S5. Cell death expected and induced by combinations based on Aurora kinase inhibitors. Cells were incubated with the indicated concentrations of the drugs for 48 + 24 hours (A,B) and 24–72 h (C). Induced cell death was determined by Annexin V-DY634 binding/7-AAD staining and flow cytometry. EXP and OBS specific cell death of each combination based on Aurora kinase inhibitors are shown. Data from 2–5 (A), 3–5 (B) and 3–7 (C) independent experiments are illustrated. Statistical analysis was performed by using two-tailed paired t-test, comparing the expected and observed cell death for each combination. * *p* < 0.05; ** *p* < 0.01; *** *p* < 0.001; **** *p* < 0.0001. File S1: Original western blots.

## Abbreviations

The following abbreviations are used in this manuscript: (D)KO, (double) knockout; (mt)ROS, (mitochondrial) reactive oxygen species; 7-AAD, 7-amino-actinomycin D; ABB, Annexin V binding buffer; ALL, acute lymphocytic leukemia; AML, acute myeloid leukemia; ASCT, autologous stem cell transplantation; BSA, bovine serum albumin; CDK, cyclin-dependent kinase; CLL, chronic lymphocytic leukemia; CPC, chromosomal passenger complex; CRISPR, clustered regularly interspaced short palindromic repeats; DCA, Dichloroacetate; DiOC_6_(3), 3,3′-dihexyloxacarbocyanine iodide; EMA, European Medicines Agency; EXP, hypothetical expected specific apoptosis; FBS, fetal bovine serum; FDA, Food and Drug Administration; GSH, glutathione; IC_50_, half-maximal inhibitory concentration; IMiDs, immunomodulatory drugs; MFI, median fluorescence intensity; MM, multiple myeloma; MOMP, mitochondrial outer membrane permeabilization; mtDNA, mitochondrial DNA; MTT, 3-(4,5-dimethylthiazol-2-yl)-2,5-diphenyltetrazolium bromide; Nec-1, Necrostatin-1; NSCLC, non-small-cell lung cancer; OBS, experimentally observed specific apoptosis; PBS, phosphate-buffered saline; PFA, paraformaldehyde; PI, propidium iodide; PS, phosphatidylserine; RIPK1, receptor-interacting serine/threonine-protein kinase 1; SAC, spindle assembly checkpoint; SA-β-gal, senescence-associated β-galactosidase; SLL, small lymphocytic lymphoma; STR, short tandem repeat; TMRE, Tetramethylrhodamine ethyl ester; ΔΨ_m_, mitochondrial transmembrane potential.
