# Supplementary material for: Alisertib and Barasertib Induce Cell Cycle Arrest and Mitochondria-Related Cell Death in Multiple Myeloma with Enhanced Efficacy Through Sequential Combination with BH3-Mimetics and Panobinostat
Source: Cancers (Basel). 2025 Jul 9;17(14):2290. doi: 10.3390/cancers17142290 (PMC12294101; doi:10.3390/cancers17142290)
Supplement: Supplementary file 1 [file cancers-17-02290-s001.zip › cancers-3703060-supplementary.pdf]

Supplementary Figure Legends

Benedi *et al*. “Targeting Aurora Kinases in multiple myeloma: Mechanisms of action and synergistic therapeutic strategies”**.**

Supplementary Figure S1. Simultaneous analysis of mitochondrial transmembrane potential and PS exposure in MM cells treated with alisertib or barasertib. Cells were treated for 72 with the indicated concentrations of AURKi, stained with TMRE and AnnexinV-DY634 and analyzed by and flow cytometry. Representative dot-plots are shown.

Supplementary Figure S 2. Sensitivity of Bax/Bak^{DKO} MM.1S cells to alisertib. Genetically modified MM cells were treated for 24 and 48 hours with increasing concentrations of alisertib (0-1 µM) and triggered cell death was assessed by Annexin V-DY634 binding/7-AAD staining and flow cytometry (n=2-7). Specific cell death is depicted and global mean and SD are represented. Two-tailed unpaired t-test statistical analysis was performed, comparing each drug concentration in the Bim-deficient lines to the infected control line. *p<0,05; **p<0,01; ***p<0,001; ****p<0,0001.

Supplementary Figure S 3. Representative histograms from flow cytometry analysis indicating the percentage of cytochrome c release into the cytosol with each treatment in MM.1S and MM.1S Bax/Bak^{DKO} cells.

Supplementary Figure S 4. Morphological changes induced by Aurora kinase inhibitors in U937 ρ^{0} cells. U937 *wild-type* and ρ^{0} cells were treated for 72 hours with increasing concentrations of alisertib and barasertib (0-5 µM). Purple arrows point to apoptotic cells, blue arrows indicate necrotic cells and black irregular shapes show spherical cell debris. Scale bar = 25 µm (U937 *wild-type* cells) and 50 µm (U937 ρ^{0} cells).

Supplementary Figure S5. Cell death expected and induced by combinations based on Aurora kinase inhibitors. Cells were incubated with the indicated concentrations of the drugs for 48 + 24 hours (**A-B**) and 24-72 hours (**C**). Induced cell death was determined by Annexin V-DY634 binding/7-AAD staining and flow cytometry. EXP and OBS specific cell death of each combination based on Aurora kinase inhibitors are shown. Data from 2-5 (A), 3-5 (B) and 3-7 (C) independent experiments are illustrated. Statistical analysis was performed by using two-tailed paired t-test, comparing the expected and observed cell death for each combination. *p<0,05; **p<0,01; ***p<0,001; ****p<0,0001.

Figure S1

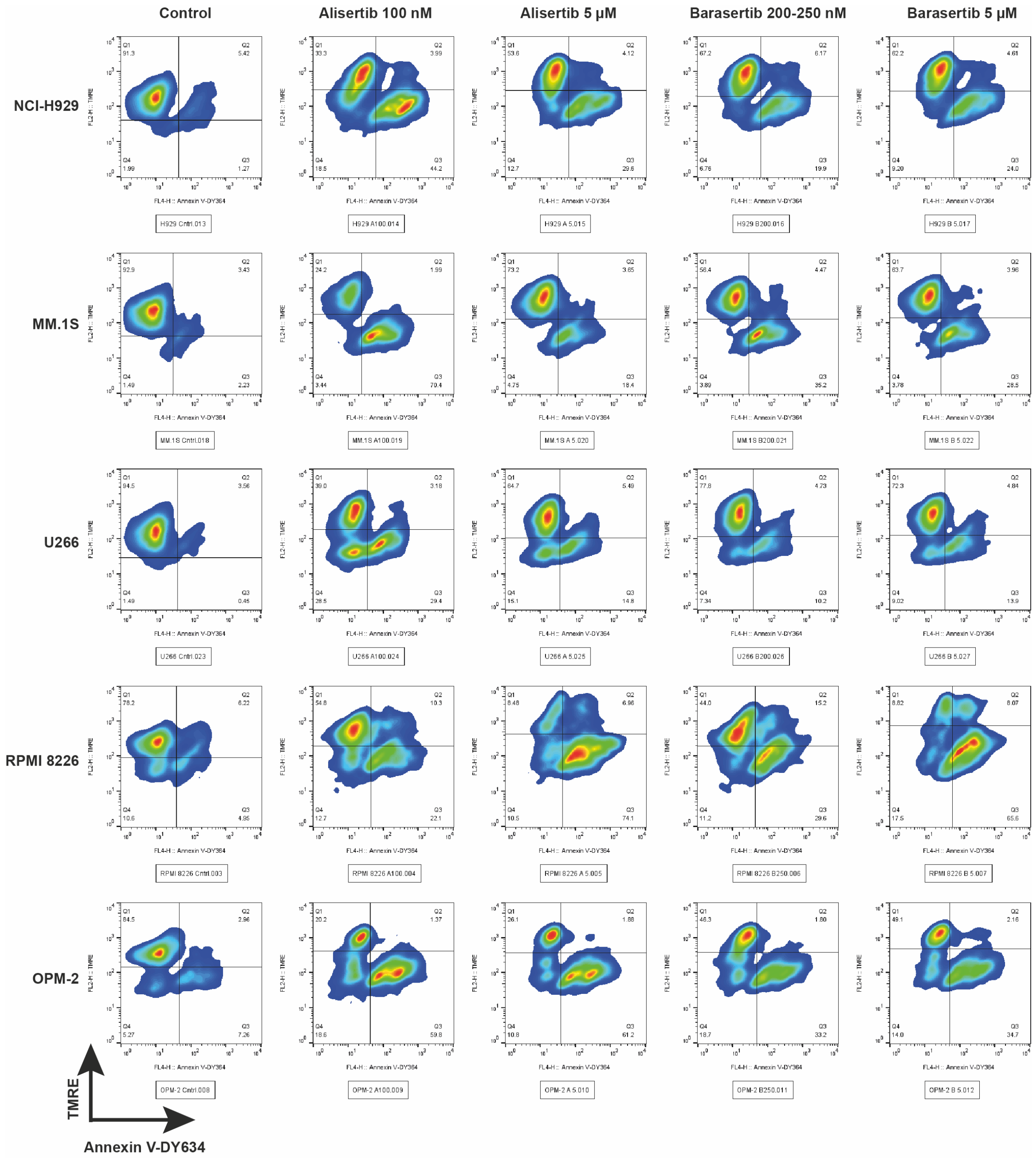
Control
Alisertib 100 nM
Alisertib 5 µM
Barasertib 200-250 nM
Barasertib 5 µM
NCI-H929
MM.1S
U266
RPMI 8226
OPM-2
TMRE
Annexin V-DY634

Figure S2

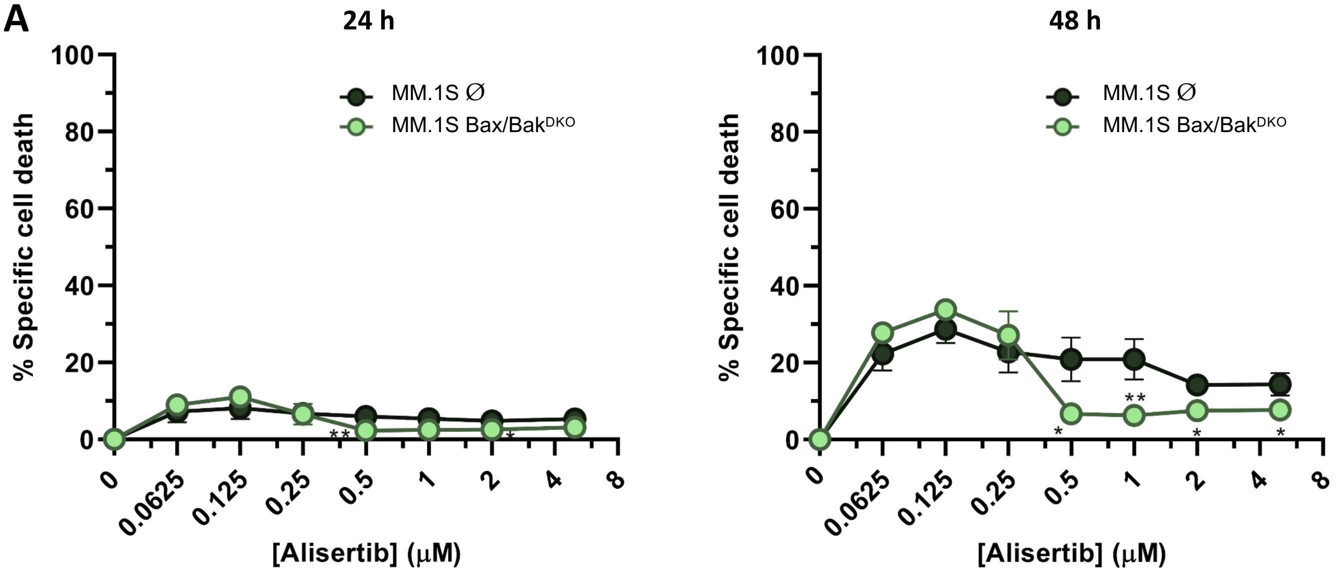
A
24 h
100
80
60
40
20
0
% Specific cell death
MM.1S Ø
MM.1S Bax/Bak^DKO
**
*
0
0.0625
0.125
0.25
0.5
1
2
4
8
[Alisertib] (μM)
48 h
100
80
60
40
20
0
% Specific cell death
MM.1S Ø
MM.1S Bax/Bak^DKO
*
**
*
*
0
0.0625
0.125
0.25
0.5
1
2
4
8
[Alisertib] (μM)

Figure S3

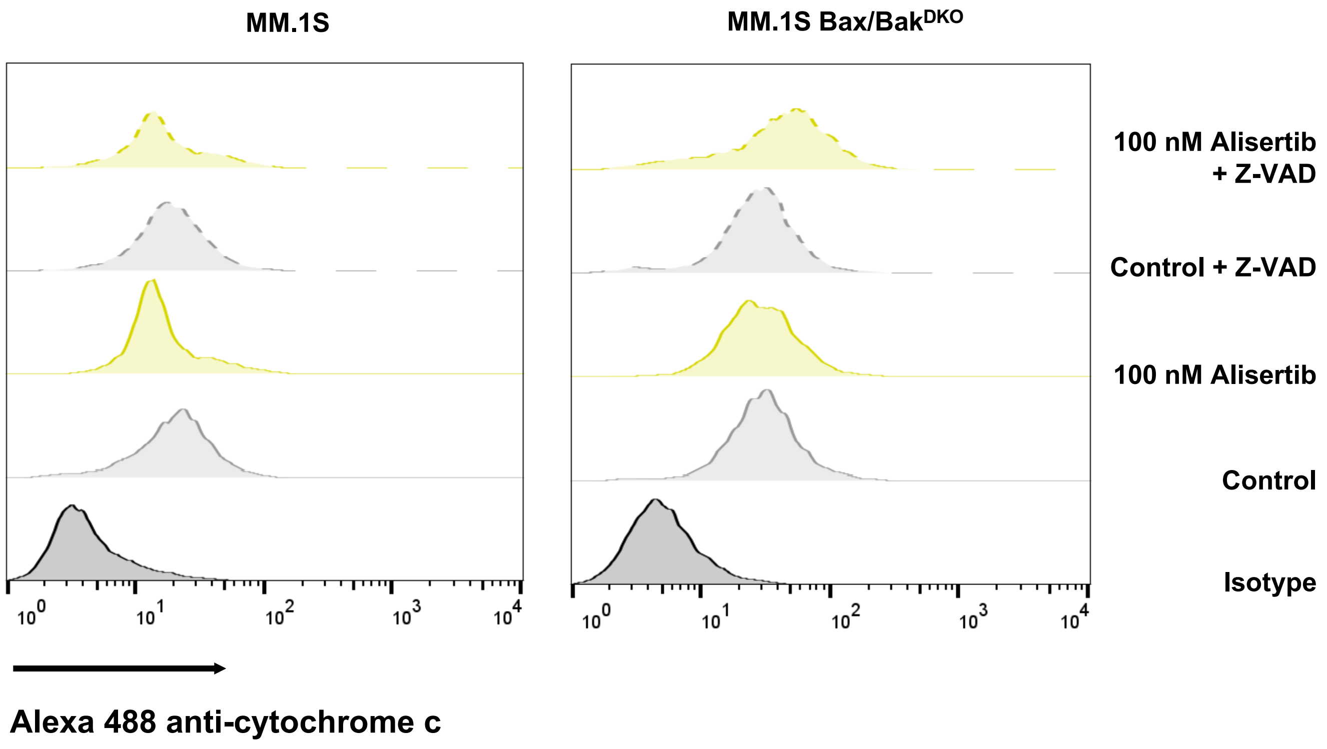

MM.1S
MM.1S Bax/Bak^{DKO}
100 nM Alisertib + Z-VAD
Control + Z-VAD
100 nM Alisertib
Control
Isotype
10^0 10^1 10^2 10^3 10^4
Alexa 488 anti-cytochrome c

Figure S4

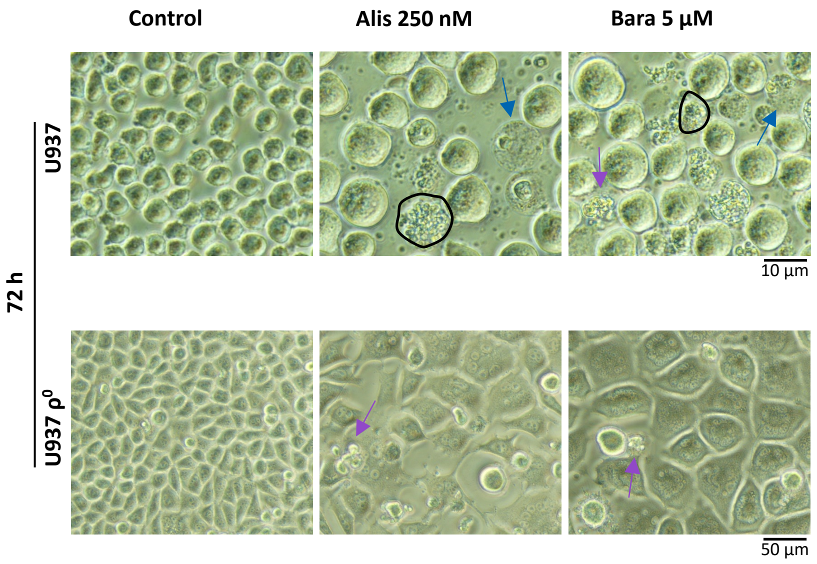

Figure S5

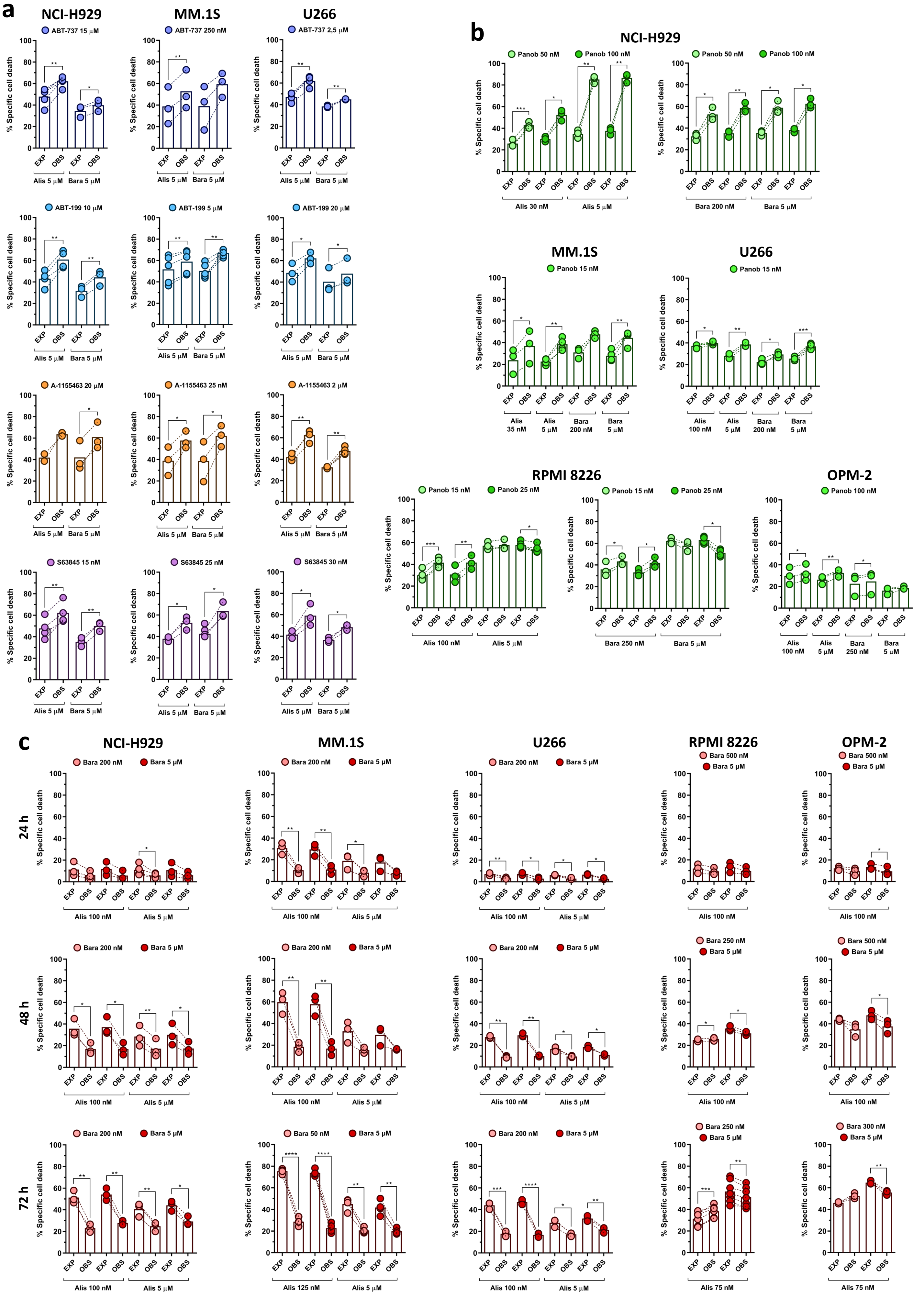

a
NCI-H929
MM.1S
U266
ABT-737 15 μM
ABT-737 250 nM
ABT-737 2,5 μM
ABT-199 10 μM
ABT-199 5 μM
ABT-199 20 μM
A-1155463 20 μM
A-1155463 25 nM
A-1155463 2 μM
S63845 15 nM
S63845 25 nM
S63845 30 nM
% Specific cell death
EXP
OBS
Alis 5 μM
Bara 5 μM
b
NCI-H929
Panob 50 nM
Panob 100 nM
Alis 30 nM
Alis 5 μM
Bara 200 nM
Bara 5 μM
MM.1S
U266
Panob 15 nM
Alis 35 nM
Alis 100 nM
Alis 5 μM
Bara 200 nM
Bara 5 μM
RPMI 8226
Panob 15 nM
Panob 25 nM
Alis 100 nM
Alis 5 μM
Bara 250 nM
Bara 5 μM
OPM-2
Panob 100 nM
Alis 100 nM
Alis 5 μM
Bara 250 nM
Bara 5 μM
c
NCI-H929
MM.1S
U266
RPMI 8226
OPM-2
24 h
48 h
72 h
Bara 200 nM
Bara 5 μM
Bara 500 nM
Bara 250 nM
Bara 50 nM
Bara 300 nM
Alis 100 nM
Alis 5 μM
Alis 125 nM
Alis 75 nM
