# Supplementary figures and images for: Alisertib and Barasertib Induce Cell Cycle Arrest and Mitochondria-Related Cell Death in Multiple Myeloma with Enhanced Efficacy Through Sequential Combination with BH3-Mimetics and Panobinostat
Source: Cancers (Basel). 2025 Jul 9;17(14):2290. doi: 10.3390/cancers17142290 (PMC12294101; doi:10.3390/cancers17142290)

Original images of Blots (Figure 3a)

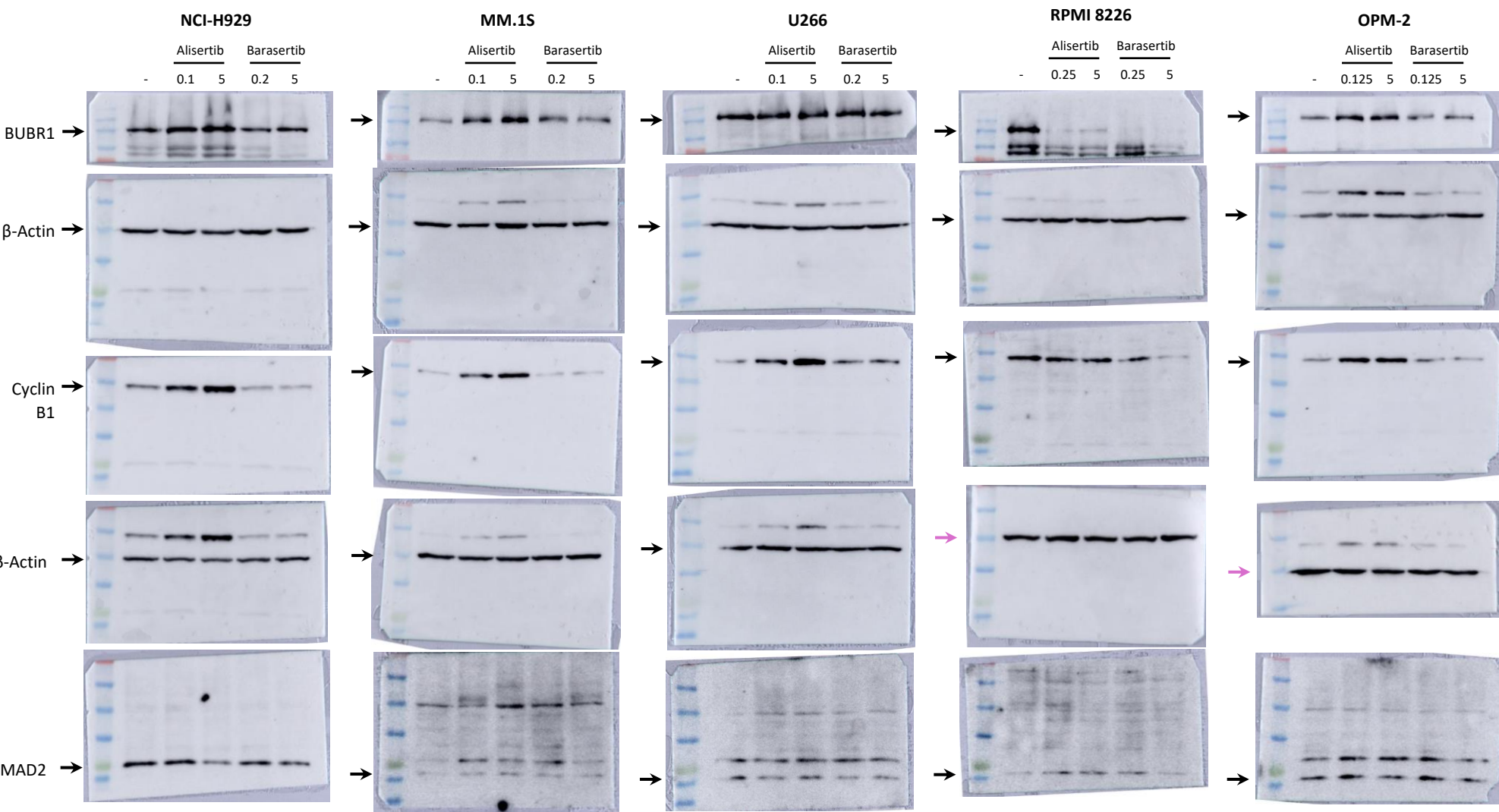

Supplement: Supplementary file 1 [file cancers-17-02290-s001.zip › cancers-3703060-File S1.pdf]
